# Role of EZH2 in Uterine Gland Development

**DOI:** 10.3390/ijms232415665

**Published:** 2022-12-10

**Authors:** Nan Ni, Frank L. Jalufka, Xin Fang, Dylan A. McCreedy, Qinglei Li

**Affiliations:** 1 Department of Veterinary Integrative Biosciences, Texas A&M University, College Station, TX 77843, USA; 2 Department of Biology, Texas A&M University, College Station, TX 77843, USA; 3 Department of Biomedical Engineering, Texas A&M University, College Station, TX 77843, USA; 4 Institute for Neuroscience, Texas A&M University, College Station, TX 77843, USA

**Keywords:** EZH2, endometrium, adenogenesis

## Abstract

Enhancer of zeste homolog 2 (EZH2) is a core component of polycomb repressive complex 2 that plays a vital role in transcriptional repression of gene expression. Conditional ablation of EZH2 using progesterone receptor (*Pgr*)-Cre in the mouse uterus has uncovered its roles in regulating uterine epithelial cell growth and stratification, suppressing decidual myofibroblast activation, and maintaining normal female fertility. However, it is unclear whether EZH2 plays a role in the development of uterine glands, which are required for pregnancy success. Herein, we created mice with conditional deletion of *Ezh2* using anti-Mullerian hormone receptor type 2 (*Amhr2*)-Cre recombinase that is expressed in mesenchyme-derived cells of the female reproductive tract. Strikingly, these mice showed marked defects in uterine adenogenesis. Unlike *Ezh2 Pgr*-Cre conditional knockout mice, deletion of *Ezh2* using *Amhr2*-Cre did not lead to the differentiation of basal-like cells in the uterus. The deficient uterine adenogenesis was accompanied by impaired uterine function and pregnancy loss. Transcriptomic profiling using next generation sequencing revealed dysregulation of genes associated with signaling pathways that play fundamental roles in development and disease. In summary, this study has identified an unrecognized role of EZH2 in uterine gland development, a postnatal event critical for pregnancy success and female fertility.

## 1. Introduction

Polycomb group genes are evolutionally conserved enforcers of epigenetic status and are implicated in cell differentiation and development [1]. Polycomb repressive complex 1 (PRC1) and PRC2 play vital roles in gene silencing through transcriptional repression [2]. The core subunits of PRC2 consist of enhancer of zeste homolog 2 (EZH2) and EZH1, SUZ12 PRC2 subunit (SUZ12), and embryonic ectoderm development (EED) [3]. The enzymatic activity of PRC2 is mediated by EZH1 and EZH2, which catalyze the trimethylation of lysine 27 of histone H3 (H3K27me3) [3]. 

EZH2 is important for numerous biological and pathological processes, including but not limited to, cell lineage commitment and carcinogenesis [4,5]. Mutation or overexpression of EZH2 occurs in multiple types of cancers, and current literature supports EZH2 as a key regulator of cancer development and a promising target for cancer therapy [5]. The involvement of EZH2 in the pathogenesis of endometrial carcinoma and endometriosis has also been reported [6,7]. It has been shown that ectopic endometrial lesions express higher levels of EZH2; downregulation of *EZH2* attenuates the migration and invasion of endometrial epithelial cells and the expression of genes associated with epithelial-mesenchymal transition [7]. Of note, both tumor-suppressive and tumor-promoting functions of EZH2 have been reported in cancer development [8,9,10,11,12,13,14].

In contrast to the unequivocal roles of EZH2 in cancer development, understanding of the function of EZH2 in the female reproductive tract is far from complete. *Ezh2* null mice are embryonically lethal [15], precluding the functional analysis of its postnatal role in the uterus. In vitro studies using human decidualizing endometrial cells support a potential role for EZH2 in endometrial decidualization, where loss of H3K27me3 is accompanied by the enrichment of acetylation at the proximal promoters of decidual marker genes [16]. In contrast, a recent study has shown that EZH2 prevents transforming growth factor β-mediated wound healing response in the mouse decidua [17], suggesting potential species-specific roles of EZH2 or differences between in vivo and in vitro model systems for decidualization.

We have reported that conditional deletion of *Ezh2* in the mouse uterus using progesterone receptor (*Pgr*)-Cre [18] results in fertility defects, endometrial hyperplasia, and stratification of uterine epithelia [19]. Increased epithelial proliferation was found using ovariectomized mice harboring *Pgr*-Cre-mediated *Ezh2* deletion [20]. Furthermore, estrogen (E2) induces EZH2 expression in uterine epithelia of wild-type mice at least partially via membrane estrogen receptor 1 (ESR1) [20]. Further transcriptomics and spatial transcriptomics analyses have revealed a potential involvement of ESR1, Wingless-type MMTV integration site (WNT), and Hippo pathways in uterine abnormalities as well as epithelial and stromal changes of gene expression in the EZH2-deficient uterus [21,22]. 

Uterine glands together with their secretions are important regulators of uterine receptivity, implantation, and embryo development [23]. During the first trimester, human endometrial secretions provide important nutrients and growth factors [24]. Uterine gland formation is a postnatal event in mice [25,26], with WNT signaling being a well-established regulator [27,28,29,30]. In this study, we created mice with conditional deletion of *Ezh2* using anti-Mullerian hormone receptor type 2 (*Amhr2*)-Cre that is expressed in mesenchyme-derived cells in the female reproductive tract [31]. Our results revealed an unrecognized role of EZH2 in postnatal uterine gland development.

## 2. Results

### 2.1. Conditional Deletion of Ezh2 Using Amhr2-Cre Recombinase Causes Defective Uterine Gland Formation

Conditional deletion of *Ezh2* was generated using *Amhr2*-Cre targeting exons 14 and 15 of *Ezh2* floxed allele (Supplementary Figure S1A). To validate the mouse model, we demonstrated that *Ezh2* conditional allele was specifically recombined (*Ezh*2^d^) in the uteri of *Ezh2 Amhr2*-Cre cKO mice, in contrast to control uteri (Supplementary Figure S1B). Consistent with this observation, *Ezh2* mRNA levels were reduced in the uteri of *Ezh2 Amhr2*-Cre cKO mice compared with controls (Supplementary Figure S1C). Reduced immunoreactive signals for EZH2 in uterine stromal cells, but not epithelial cells, of *Ezh2 Amhr2*-Cre cKO mice were also found in comparison with age-matched controls (Supplementary Figure S1D–G).

**Figure 1:**
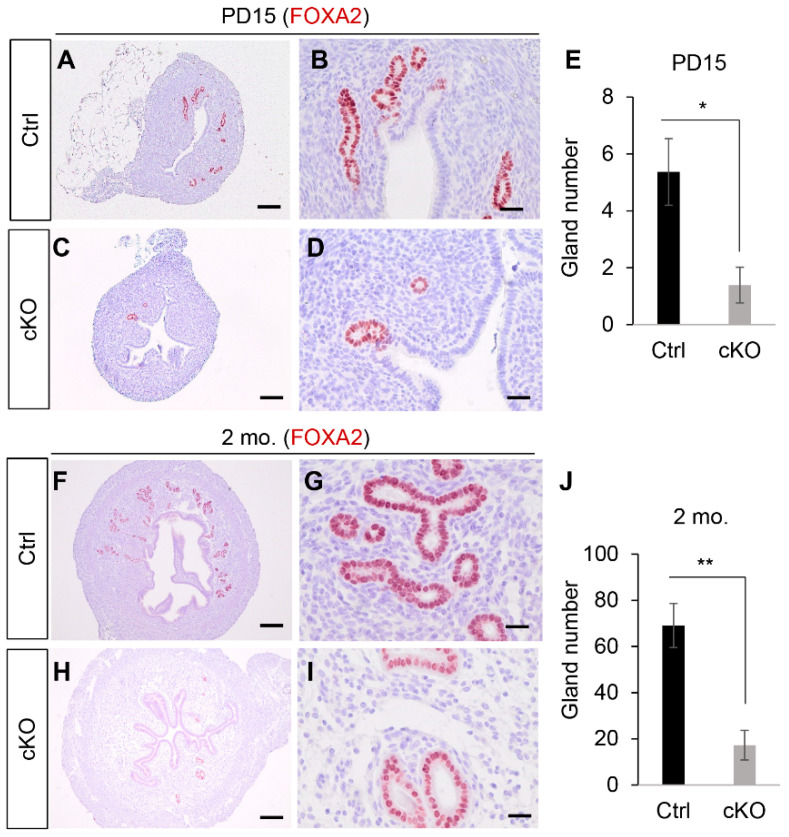
Glandular defects of the uterus in *Ezh2 Amhr2*-Cre cKO mice. (**A**–**D**) Immunostaining of FOXA2 using uteri from control and *Ezh2 Amhr2*-Cre cKO mice at PD15. Panels (**B**,**D**) are higher power images for panels (**A**,**C**), respectively. Scale bar = 25 µm (**B**,**D**) and 100 µm (**A**,**C**). At least three animals for each genotype were examined. (**E**) Quantification of the number of uterine glands using control and *Ezh2 Amhr2*-Cre cKO mice at PD15. *n* = 3. Data are mean ± s.e.m. * *p* < 0.05. (**F**–**I**) Immunostaining of FOXA2 using uteri from control and *Ezh2 Amhr2*-Cre cKO mice around 2 months of age. Panels (**G**,**I**) are higher power images for panels (**F**,**H**), respectively. Scale bar = 25 µm (**G**,**I**) and 250 µm (**F**,**H**). At least three animals for each genotype were examined. (**J**) Quantification of the number of uterine glands using control and *Ezh2 Amhr2*-Cre cKO mice around 2 months of age. *n* = 5. Data are mean ± s.e.m. ** *p* < 0.01.

Immunostaining of actin alpha 2, smooth muscle, aorta (ACTA2), vimentin (VIM), and keratin 8 (KRT8) was next performed to identify the respective myometrial, stromal, and epithelial compartments of the uterus at PD15 when the basic configuration of the uterus is established. Results showed the formation of all three uterine compartments in *Ezh2 Amhr2*-Cre cKO mice (Supplementary Figure S2A–H). However, KRT8-positive uterine glands were scarce within the endometrium of *Ezh2 Amhr2*-Cre cKO mice (Supplementary Figure S2F) compared with controls (Supplementary Figure S2E). Immunostaining of FOXA2, a uterine gland-specific marker, and the uterine morphometrics analysis demonstrated reduced abundance of uterine glands in *Ezh2 Amhr2*-Cre cKO mice at PD15 (Figure 1A–E). To determine whether this defect persisted into adulthood, we examined FOXA2-immunostained uterine sections from control and *Ezh2 Amhr2*-Cre cKO mice around 2 months of age. Similarly, less uterine glands were found in *Ezh2 Amhr2*-Cre cKO mice (Figure 1F–J). To determine if there was a potential prenatal effect of *Amhr2*-Cre expression in the Mullerian duct [32], we examined the histology of the uterus of the newborn mice. H.E. staining showed differentiation of both stromal and epithelial compartments in *Ezh2 Amhr2*-Cre cKO mice at birth, similar to age-matched controls (Supplementary Figure S3A,B). Furthermore, circular muscle layers were apparent in both control and *Ezh2 Amhr2*-Cre cKO mice at PD7 (Supplementary Figure S3C,D). Thus, results indicate that conditional deletion of *Ezh2* using *Amhr2*-Cre impairs postnatal uterine gland formation.

**Figure 2:**
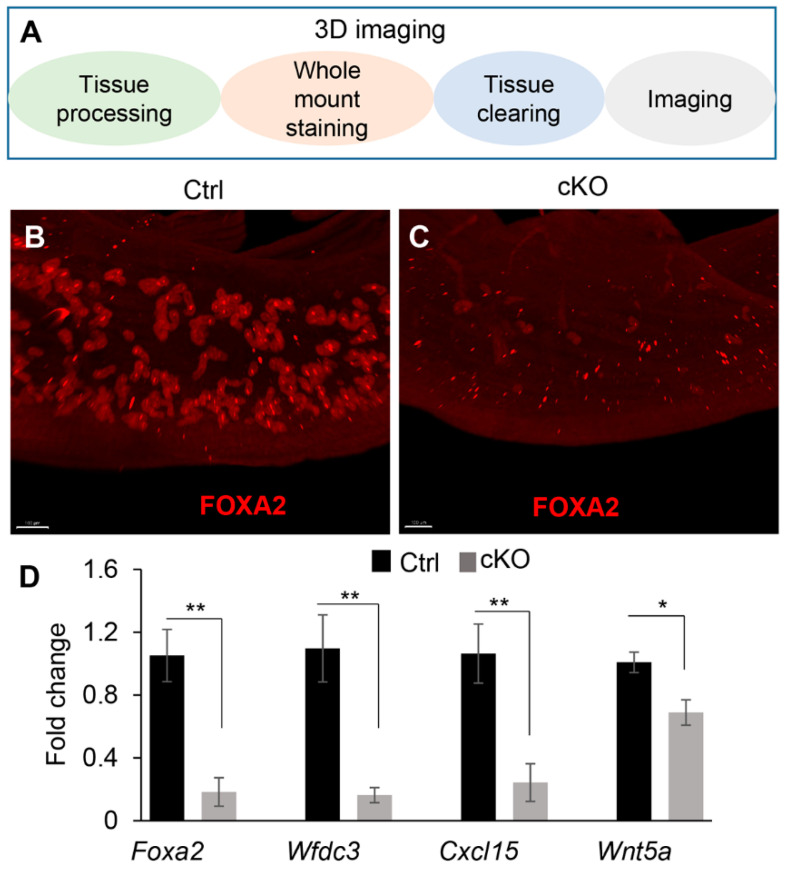
Tridimensional imaging showing defective uterine gland development in *Ezh2 Amhr2*-Cre cKO mice. (**A**) Primary steps for 3D imaging analysis. (**B**,**C**) Tridimensional view of uteri from control and *Ezh2 Amhr2*-Cre cKO mice by whole-mount staining of FOXA2 at 1 month of age. Three independent samples for each genotype were examined. Scale bar = 200 µm. (**D**) qRT-PCR analysis of uterine gland- and adenogenesis-associate genes using uteri from 1-month-old control and *Ezh2 Amhr2*-Cre cKO mice. *n* = 5 for each genotype. Data are mean ± s.e.m. * *p* < 0.05 and ** *p* < 0.01.

To substantiate the finding of uterine gland defects in *Amhr2*-Cre cKO mice, we performed whole-mount immunofluorescent staining of FOXA2 using uteri from *Ezh2 Amhr2*-Cre cKO mice and controls at 1 month of age (Figure 2A). Tridimensional (3D) imaging showed substantially reduced FOXA2-positive glands in the uteri of *Ezh2 Amhr2*-Cre cKO mice compared with controls (Figure 2B,C). Consistent with the observed uterine gland deficiency, transcript levels of uterine gland- and adenogenesis-associated genes (i.e., *Foxa2*, *Wfdc3*, *Cxcl15*, and *Wnt5a*) were decreased in the uteri of *Ezh2 Amhr2*-Cre cKO mice at this stage (Figure 2D). Reduced expression of most of these genes was also detectable at PD10, an earlier timepoint of uterine gland development (Supplementary Figure S4).

As *Amhr2*-Cre is also expressed in the ovary and oviduct [31,33], we performed H.E. staining to determine whether *Ezh2 Amhr2*-Cre cKO mice had altered ovarian and oviductal histology. Morphologically normal ovaries and oviducts were found in *Ezh2 Amhr2*-Cre cKO mice, with the presence of ovarian follicles at various developmental stages (Supplementary Figure S5A,B,D,E). Additionally, both EZH2 immunostaining of ovarian granulosa cells (Supplementary Figure S5C,F) and *Ezh2* mRNA levels of the ovary (Supplementary Figure S5G) were comparable between control and *Ezh2 Amhr2*-Cre cKO mice. Blastocysts could be recovered from the uteri of both control and *Ezh2 Amhr2*-Cre cKO mice at E3.5 (Supplementary Figure S5H), indicating that ovulation occurred in *Ezh2 Amhr2*-Cre cKO mice. Furthermore, hormone analysis showed that serum levels of P4, E2, FSH, and LH were not significantly altered in *Ezh2 Amhr2*-Cre cKO mice at 3 months (Supplementary Figure S5I–L) and/or E8.5 (Supplementary Figure S5M–O) compared with corresponding controls. These findings suggest that ovarian function was not impaired in *Ezh2 Amhr2*-Cre cKO mice.

Taken together, these results demonstrated impaired postnatal uterine adenogenesis in *Ezh2 Amhr2*-Cre cKO mice and revealed an important role of EZH2 in uterine gland development.

### 2.2. Conditional Deletion of Ezh2 Using Amhr2-Cre Does Not Cause Basal-like Cell Phenotype in the Uterus

**Figure 3:**
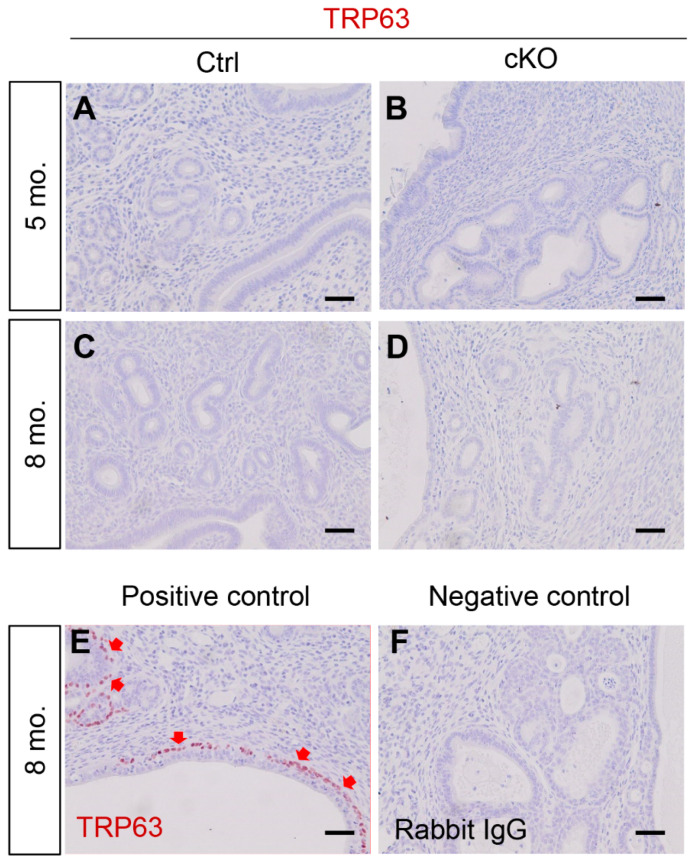
Basal-like cells do not develop in *Ezh2 Amhr2*-Cre cKO mice. (**A**–**D**) Immunostaining of TRP63 using uteri from control and *Ezh2 Amhr2*-Cre cKO mice at 5 and 8 months of age. At least three animals for each genotype were examined. (**E**,**F**) The respective positive and negative control using uterine sections from an 8-month-old *Ezh2 Pgr*-Cre cKO mouse. Red arrows indicate some TRP63-positive cells surrounding the epithelial compartment. Scale bar = 50 µm (**A**–**F**).

We previously reported that conditional ablation of EZH2 in the mouse uterus using *Pgr*-Cre leads to basal cell-like phenotype in the uterus [19]. Herein, we asked the question of whether basal-like cells developed in *Ezh2 Amhr2*-Cre cKO mice. Our results showed that basal cell marker TRP63 was not expressed in uterine epithelia of *Ezh2 Amhr2*-Cre cKO mice at 5 and 8 months of age (Figure 3A–D). These timepoints were selected because epithelial stratification is culminated in the uteri of aged *Ezh2 Pgr*-Cre cKO mice [19]. As expected, positive controls using uteri from 8-month-old nulliparous *Ezh2 Pgr*-Cre cKO mice showed clear signals of TRP63-postive cells (Figure 3E). Negative control is depicted in Figure 3F. Additionally, we examined the expression of KRT14 that is expressed in stratified epithelia. Similarly, KRT14 immunoreactive signals were detectable in the uteri of *Ezh2 Pgr*-Cre cKO mice but not *Ezh2 Amhr2*-Cre cKO mice (Supplementary Figure S6). Current results together with our earlier report [19] suggest cell-type and/or developmental stage-specific roles of EZH2 in the female reproductive tract.

### 2.3. Transcriptomic Profiling to Identify Molecular Changes Associated with Uterine Gland Abnormalities in Ezh2 Conditionally Deleted Mice

**Figure 4:**
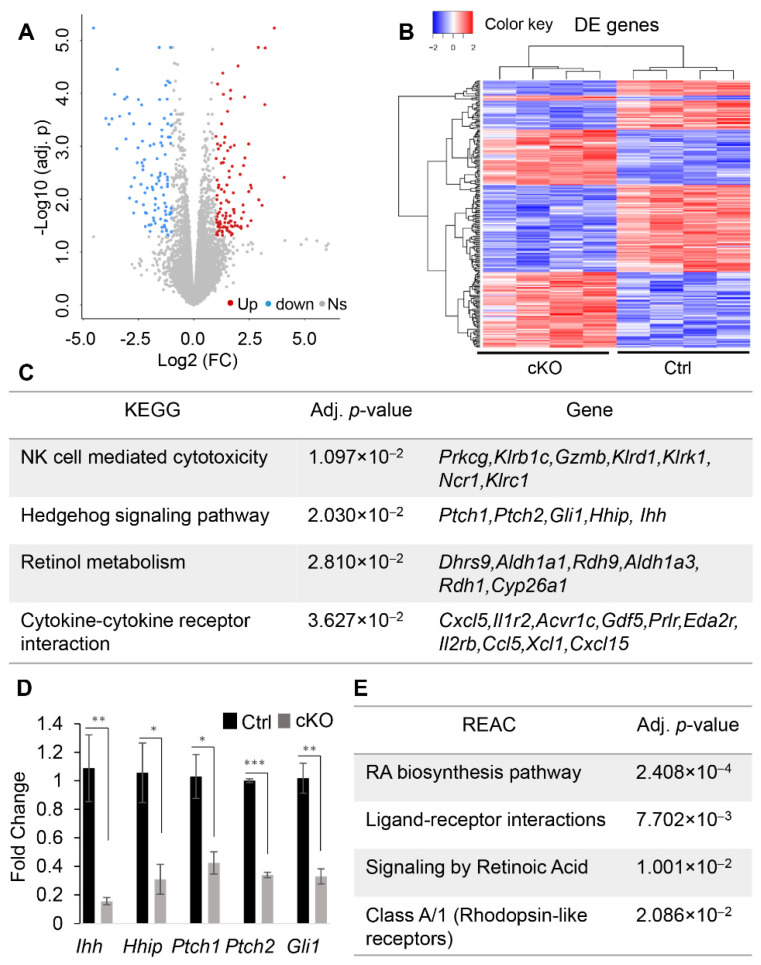
Transcriptomic profiling using RNA-seq to identify molecular changes in *Ezh2* conditionally deleted uteri. (**A**) Volcano plot for genes identified by RNA-seq using uteri from control and *Ezh2 Amhr2*-Cre cKO mice at PD10. Ns, not significant. (**B**) Heatmap depicting uterine DE genes between control and *Ezh2 Amhr2*-Cre cKO mice at PD10. (**C**) Significantly enriched pathways in the uteri of *Ezh2 Amhr2*-Cre cKO mice identified by g:Profiler tool using KEGG database. (**D**) Validation of DE genes related to hedgehog signaling using uteri from *Ezh2 Amhr2*-Cre cKO mice and controls at PD10. *n* = 4. Data are mean ± s.e.m. * *p* < 0.05, ** *p* < 0.01, and *** *p* < 0.001. (**E**) Significantly enriched pathways in the uteri of *Ezh2 Amhr2*-Cre cKO mice identified by g:Profiler analysis using REAC database.

To determine the molecular changes underlying uterine gland defects, RNA-seq was conducted using uterine tissues from *Ezh2 Amhr2*-Cre cKO mice and controls at PD10. Transcriptomic profiling revealed 212 DE genes in *Ezh2 Amhr2*-Cre cKO mice compared with controls (Figure 4A,B and Supplementary Table S1). Bioinformatic analysis was performed using g:Profiler, which identified 4 significantly enriched KEGG (Kyoto Encyclopedia of Genes and Genomes) pathways including NK cell mediated cytotoxicity, hedgehog signaling, retinol metabolism, and cytokine-cytokine receptor interaction (Figure 4C). The implication of altered NK cell-mediated cytotoxicity in *Ezh2 Amhr2*-Cre cKO mice is unclear. Hedgehog signaling plays important roles in development and disease [34]. The pathway contains hedgehog ligands, Patched (PTCH) receptors, Smoothened (SMO) protein, and GLI transcription factors. In the uteri of *Ezh2 Amhr2*-Cre cKO mice, expression of several key genes associated with hedgehog signaling including Indian hedgehog (*Ihh*), *Ptch1*, *Ptch2*, *Gli1*, and hedgehog-interacting protein (*Hhip*) was reduced compared with controls (Figure 4C). Using qRT-PCR, we verified altered expression of these genes in the uteri of *Ezh2 Amhr2*-Cre cKO mice (Figure 4D).

**Figure 5:**
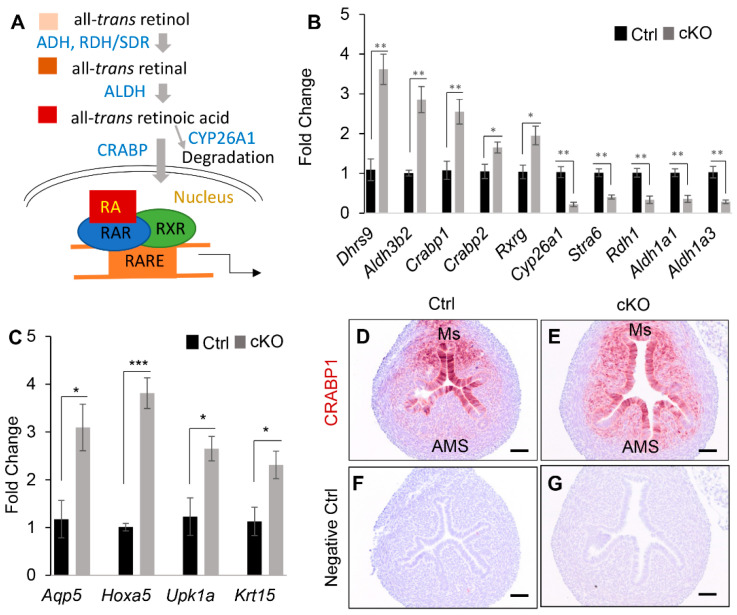
Dysregulation of RA signaling in the uteri of *Ezh2 Amhr2*-Cre cKO mice at PD10. (**A**) Schematic representation of RA signaling. All-trans retinol is oxidized by ADH or RDH/SDR to all-trans retinal, which is further oxidized to all-trans RA via ALDH. RA can be transported to the nucleus by CRABP and induce signaling events via interacting with its nuclear receptors. RA can also be degraded by CYP26A1. (**B**,**C**) qRT-PCR analysis of RA signaling-associated genes using uteri from control and *Ezh2 Amhr2*-Cre cKO mice at PD10. *n* = 4. Data are mean ± s.e.m. * *p* < 0.05, ** *p* < 0.01, and *** *p* < 0.001. (**D**–**G**) Immunostaining of CRABP1 in the uteri of *Ezh2 Amhr2*-Cre cKO mice and controls at PD10. At least three animals for each genotype were examined. Scale bar = 50 µm (**D**–**G**).

An interesting finding is the dysregulation of retinoic acid (RA) signaling in *Ezh2* conditionally deleted uteri, because RA has been involved in the regulation of epithelial duct morphogenesis and gland development in other organs [35,36]. In line with the enrichment of retinol metabolism in KEGG pathways (Figure 4C), reactome (REAC) pathways were also enriched for RA biosynthesis pathway and RA signaling (Figure 4E). In this pathway, retinol is oxidized by dehydrogenase/reductase (SDR family), dehydrogenase (ADH), and retinol dehydrogenase (RDH). Oxidized RA binds with RA receptor (RAR), forming a heterodimer with the retinoid X receptor (RXR). RAR/RXR then bind to RA-responsive elements (RARE) to regulate target gene expression [37] (Figure 5A). qRT-PCR analysis verified altered expression of several RA signaling components and substantiated the RNA-seq results by demonstrating upregulation of a number of RA-related genes in the uteri of *Ezh2 Amhr2*-Cre cKO mice, including SDR family member 9 (*Dhrs9*)*, Aldh3b2,* cellular retinoic acid binding protein I (*Crabp1*)*, Crabp2*, and retinoid X receptor gamma (*Rxrg*) (Figure 5B). Because *Dhrs9* encodes an enzyme involved in the first step of RA metabolism [38], *Crabp1* and *Crabp2* facilitate nuclear transport of RA and the binding of RA with its cognate receptor complex, and *Rxrg* is a nuclear receptor of RA [37], upregulation of these genes in *Ezh2 Amhr2*-Cre cKO mice suggests potentiated RA signaling. This postulation was supported by downregulation of cytochrome P450, family 26, subfamily a, polypeptide 1 (*Cyp26a1*) which encodes an RA degradation enzyme [37] and upregulation of aquaporin 5 (*Aqp5*) [39], homeobox A5 (*Hoxa5*) [40], uroplakin 1A (*Upk1a*) [41], and keratin 15 (*Krt15*) [42], downstream targets of RA signaling (Figure 5C).

As CRABP1 is important for RA binding and transport, we further examined its expression using immunohistochemistry. Interestingly, CRABP1 immunoreactive signals were increased in uterine stroma of *Ezh2 Amhr2*-Cre cKO mice, particularly at the antimesometrial region where uterine glands form [43] (Figure 5D–G). Thus, dysregulation of CRABP1-dependent RA signaling in the antimesometrial endometrium may interfere with uterine gland development in *Ezh2 Amhr2*-Cre cKO mice. 

Despite the aforementioned evidence supporting increased RA signaling in *Ezh2 Amhr2*-Cre cKO uteri at PD10, downregulation of genes encoding retinol dehydrogenase 1 (RDH1; an enzyme oxidizing retinol), stimulated by retinoic acid gene 6 (STRA6; a protein responsible for vitamin A uptake) [44], RDH9, aldehyde dehydrogenase family 1, subfamily A1 (ALDH1A1), and ALDH1A3 was also found (Figure 4C and Figure 5B, and Supplementary Table S1). The impact of altered expression of these genes on RA signaling activity remains unknown. Collectively, the RNA-seq data revealed a potential link between dysregulated RA signaling and defective uterine adenogenesis in *Ezh2 Amhr2*-Cre cKO mice. 

### 2.4. Conditional Deletion of Ezh2 Leads to Impaired Uterine Function

**Figure 6:**
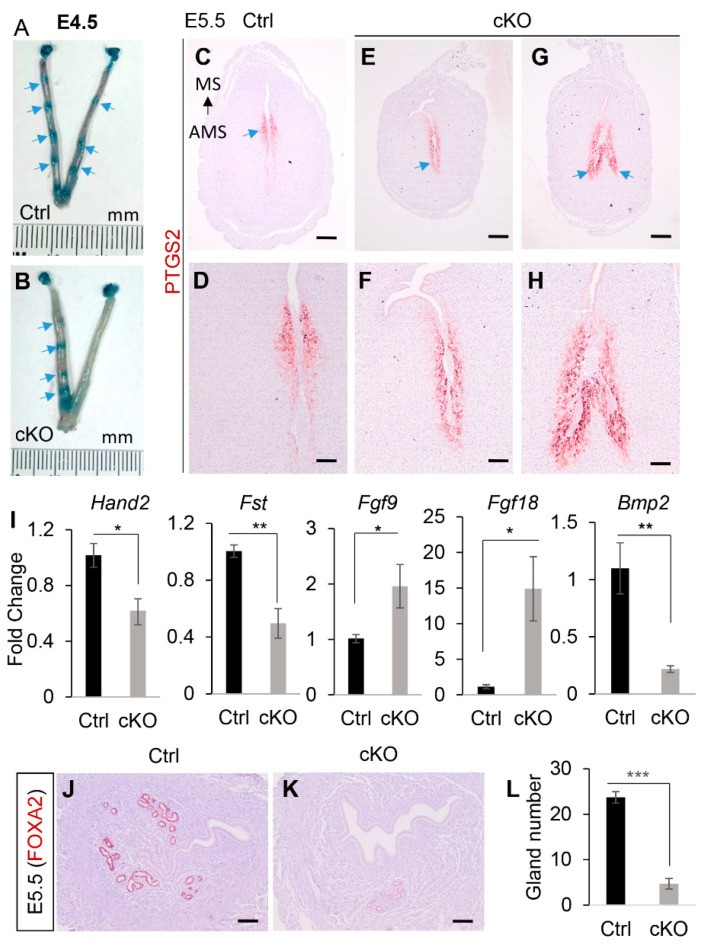
Abnormal implantation in *Ezh2 Amhr2*-Cre cKO mice. (**A**,**B**) Chicago blue injection of control and *Ezh2 Amhr2*-Cre cKO mice at E4.5. Arrows indicate implantation sites. (**C**–**H**) Immunostaining of PTGS2 using uteri from control and *Ezh2 Amhr2*-Cre cKO mice at E5.5. Panels (**D**,**F**,**H**) are higher power images for panels (**C**,**E**,**G**), respectively. Arrows indicate the location of PTGS staining. MS, mesometrial side. AMS, antimesometrial side. Scale bar = 100 µm (**D**,**F**,**H**) and 250 µm (**C**,**E**,**G**). (**I**) qRT-PCR analysis of *Hand2*, *Fst*, *Fgf9*, *Fgf18*, and *Bmp2* using implantation sites from control and *Ezh2 Amhr2*-Cre cKO mice at E4.5. *n* = 6. Data are mean ± s.e.m, * *p* < 0.05 and ** *p* < 0.01. (**J**,**K**) Reduced numbers of FOXA2-positive uterine glands in the inter-implantation sites of *Ezh2 Amhr2*-Cre cKO mice at E5.5. At least three animals for each genotype were examined. Scale bar = 100 µm (**J**,**K**). (**L**) Quantification of the number of uterine glands. *n* = 3. Data are mean ± s.e.m, *** *p* < 0.001.

As uterine glands are important for embryo implantation [45], we first examined whether implantation was affected in *Ezh2 Amhr2*-Cre cKO mice. Chicago blue injection showed impaired implantation in *Ezh2 Amhr2*-Cre cKO mice at E4.5 (Figure 6A,B). The implantation defects were supported by the expression pattern of prostaglandin-endoperoxide synthase 2 (PTGS2), which normally shifts from the antimesometrial pole at 4–5 days post-coitum (dpc) to the mesometrial pole around 6 dpc [46]. As anticipated, PTGS2 was localized to the mesometrial pole of control mice at E5.5 (blue arrow; Figure 6C,D). However, it remained in the antimesometrial side of *Ezh2 Amhr2*-Cre cKO mice (blue arrow; Figure 6E–H). Abnormal uterine epithelial folds were also observed in *Ezh2 Amhr2*-Cre cKO mice (Figure 6G,H). These findings suggest impaired/delayed implantation in *Ezh2 Amhr2*-Cre cKO mice.

Downregulation of implantation-associated genes, *Hand2* [47] and *Fst* [48], was found in implantation sties of *Ezh2 Amhr2*-Cre cKO mice compared with controls (Figure 6I). Increased expression of *Fgf9* and *Fgf18* has been associated with implantation defects in mice with conditional deletion of bone morphogenetic protein receptor type 1A (*Bmpr1a*) [49]. Interestingly, *Fgf9* and *Fgf18* transcript levels were also elevated in *Ezh2* conditionally deleted implantation sites (Figure 6I). *Bmp2*, a critical gene involved in decidualization, was downregulated in *Ezh2 Amhr2*-Cre cKO mice (Figure 6I), suggesting abnormal decidualization. To determine whether uterine gland deficiency persisted in the pregnant uterus, immunostaining of FOXA2 was performed using E5.5 interimplantation sites from both control and *Ezh2 Amhr2*-Cre cKO mice. Indeed, the numbers of uterine glands were declined in *Ezh2 Amhr2*-Cre cKO mice compared with controls (Figure 6J–L), suggesting a link between uterine gland deficiency and impaired pregnancy.

**Figure 7:**
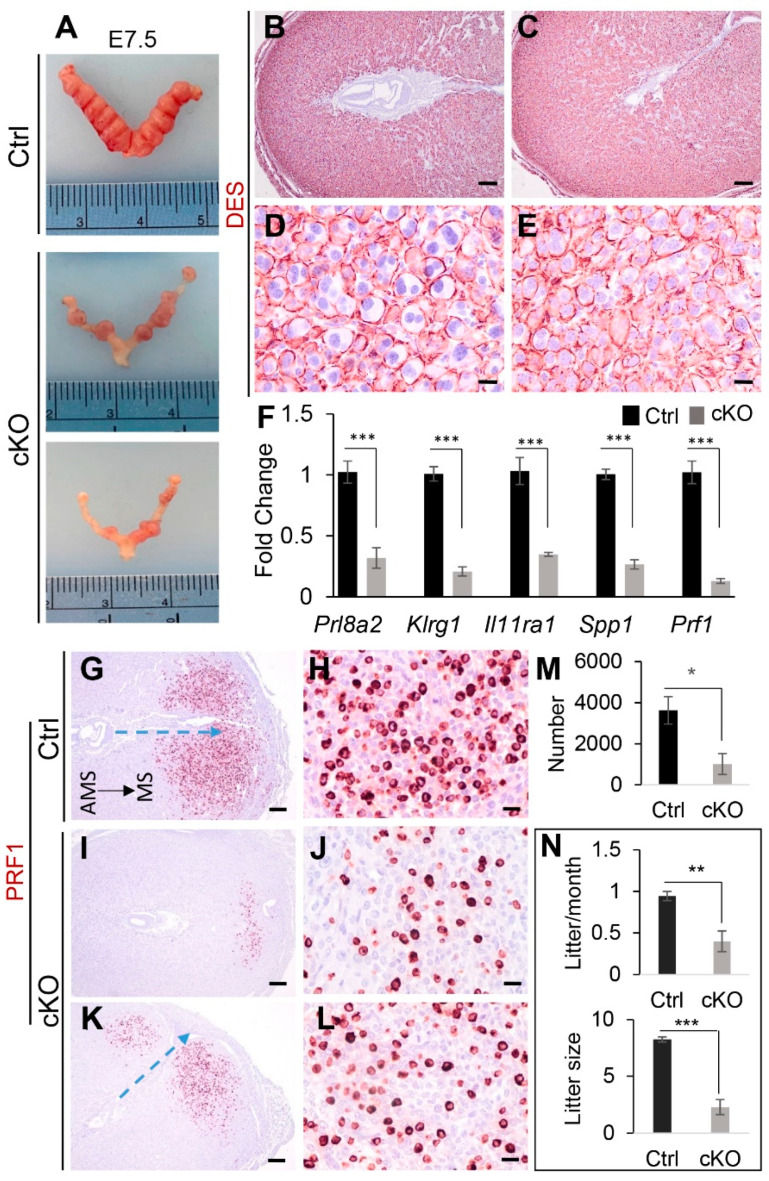
Impaired embryo development and decidual integrity in *Ezh2 Amhr2*-Cre cKO mice. (**A**) Embryo development in control and *Ezh2 Amhr2*-Cre cKO mice at E7.5. (**B**–**E**) Immunostaining of DES using uteri from control and *Ezh2 Amhr2*-Cre cKO mice at E6.5. At least three animals for each genotype were examined. Panels (**D**,**E**) are higher power images for panels (**B**,**C**), respectively. Scale bar = 20 µm (**D**,**E**) and 200 µm (**B**,**C**). (**F**) qRT-PCR analysis of *Prl8a2*, *Klrg1*, *Il11ra1*, *Spp1*, and *Prf1* mRNA expression using implantation sites from control and *Ezh2 Amhr2*-Cre cKO mice at E7.5. *n* = 6. Data are mean ± s.e.m, ***p < 0.001. (**G**–**L**) Immunostaining of PRF1 using implantation sites from control and *Ezh2 Amhr2*-Cre cKO mice at E7.5. Panels (**I**–**L**) represent two implantation sites from different *Ezh2 Amhr2*-Cre cKO mice. Panels (**H**,**J**,**L**) are higher power images for panels (**G**,**I**,**K**), respectively. At least three animals for each genotype were examined. MS, mesometrial side. AMS, antimesometrial side. Dashed blue arrows indicate embryonic-uterine orientation. Scale bar = 200 µm (**G**,**I**,**K**) and 20 µm (**H**,**J**,**L**). (**M**) Qualification of the number of PRF1-positive cells in control and *Ezh2 Amhr2-Cre* cKO mice at E7.5. *n* = 6. Data are mean ± s.e.m. * *p* < 0.05. (**N**) Subfertility in *Ezh2 Amhr2*-Cre cKO mice during a 3-month breeding trial. *n* = 5–6. Data are mean ± s.e.m. ** *p* < 0.01 and *** *p* < 0.001.

Next, we performed timed-mating to examine embryo development during early pregnancy event. As expected, defects of embryo development were found in *Ezh2 Amhr2*-Cre cKO mice, including reduced embryo size and frequently observed embryo degeneration (Figure 7A). Because uterine gland deficiency negatively impacts decidualization [45], we postulated that decidual development would be compromised in *Ezh2 Amhr2*-Cre cKO mice. Thus, we examined the expression pattern of desmin (DES) at the antimesometrial side of the uterus, which links to decidual cell differentiation during early pregnancy [50]. It was found that defective embryo development was associated with altered DES expression pattern during early pregnancy. While the periphery of mature decidual cells in the antimesometrial region of control mice was positive for DES, perinuclear DES staining and small decidual cells could be found in *Ezh2 Amhr2*-Cre cKO mice in the corresponding decidual region (Figure 7B–E). This observation was consistent with reduced expression of a decidual marker gene, *Prl8a2*, in the decidua of *Ezh2 Amhr2*-Cre cKO mice (Figure 7F). 

It is generally believed that uNK cells play important roles in pregnancy [51]. Conditional deletion of *Ezh2* using *Pgr*-Cre showed reduced numbers of uNK cells in the pregnant uterus [19]. Using qRT-PCR analysis, we found that transcript levels of uNK cell differentiation-associated genes including *Klrg1*, *Il11ra*, secreted phosphoprotein 1 (*Spp1*), and *Prf1* were downregulated in the *Ezh2 Amhr2*-Cre cKO decidua versus controls at E7.5 (Figure 7F). Next, we performed immunostaining of PRF1 using implantation sites from control and *Ezh2 Amhr2*-Cre cKO at E7.5 to further examine whether the abundance of PRF1-positive cells was altered in *Ezh2 Amhr2*-Cre cKO mice (Figure 7G–L). While abundant PRF1-expressing cells were visualized at the decidual basalis region of control mice (Figure 7G,H), the numbers appeared to be reduced in the uteri of *Ezh2 Amhr2*-Cre cKO mice (Figure 7I–L). It was interesting to note that the embryonic-uterine orientation of some implantation sites from *Ezh2 Amhr2*-Cre cKO mice was altered compared with that of controls (blue arrows; Figure 7G,K). Quantitative analysis showed that the numbers of PRF1-positive cells were significantly decreased in *Ezh2 Amhr2*-Cre cKO mice (Figure 7M). Reduction of PRF1-positive cell numbers was also found in the uteri of *Ezh2 Amhr2*-Cre cKO mice at E6.5 (Supplementary Figure S7). In addition, more PAS-positive uNK cells with cytoplasmic granules were observed in the decidua of control mice versus *Ezh2 Amhr2*-Cre cKO mice at E7.5 (Supplementary Figure S8). These findings suggest reduced abundance of uNK cells in the uteri of *Ezh2 Amhr2*-Cre cKO mice. Consistent with impaired uterine function, *Ezh2 Amhr2*-Cre cKO mice demonstrated reduced fertility during a 3-month breeding trial (Figure 7N). Thus, conditional deletion of *Ezh2* impairs the decidual integrity. These findings reinforce the importance of EZH2 in uterine development and pregnancy.

## 3. Discussion

Uterine glands provide nutrients for embryo development during pregnancy, and impaired uterine gland function has been associated with pregnancy complications including miscarriage and preeclampsia [23]. A recent demonstration of FOXA2 networks in the human endometrium provides mechanistic insights into the functional role of uterine glands in pregnancy [52]. A major finding of the present study is the role of EZH2 in uterine gland development. Reduced numbers of uterine glands were also found in the inter-implantation sites at E5.5, suggesting that impaired pregnancy events in *Ezh2 Amhr2*-Cre cKO mice are at least partially attributed to uterine gland deficiency. Despite the importance of uterine glands in pregnancy, a threshold number of uterine glands appears to be sufficient in pregnancy maintenance [25]. A reduction of approximately 30% of uterine glands does not appear to have an apparent effect on female fertility [25]. This finding may explain the observation of embryo implantation and decidualization that still occurred in *Ezh2 Amhr2*-Cre cKO mice containing significantly decreased numbers of uterine glands.

Our studies revealed distinct uterine phenotypes upon conditional deletion of *Ezh2* using *Amhr2*-Cre versus *Pgr*-Cre. We found that *Ezh2 Pgr*-Cre cKO mice, but not *Ezh2 Amhr2*-Cre cKO mice, develop a basal cell-like phenotype [19]. On the other hand, defective adenogenesis was prominent only in *Ezh2 Amhr2*-Cre cKO mice. The exact reasons for the observed phenotypic differences are unclear. As *Amhr2*-Cre is not expressed in the epithelial compartment and *Ezh2 Amhr2*-Cre cKO mice did not show basal-like cell differentiation, it is conceivable that the basal-like cell phenotype observed in *Ezh2 Pgr*-Cre cKO mice [19] might be attributed to the loss of epithelial EZH2. Then, we raised the question of why ablation of EZH2 using *Pgr*-Cre does not lead to defects in uterine adenogenesis [19]. It has been well-established that mouse uterine glands form postnatally when the luminal epithelium invaginates and branches into the endometrium [53]. Of note, *Pgr*-Cre activity appears to be limited in uterine stroma during uterine adenogenesis [18]. In contrast, the activity of *Amhr2*-Cre is detectable in the Müllerian duct mesenchyme as early as 13.5 dpc [31]. Thus, one possible explanation for the lack of uterine gland defects in *Ezh2 Pgr*-Cre cKO mice is the late onset of Cre expression or low Cre expression in the stroma during adenogenesis. However, the possibility cannot be excluded that simultaneous reduction of both epithelial and mesenchymal EZH2 using *Pgr*-Cre may lead to a distinct phenotypic outcome than conditional deletion of mesenchymal *Ezh2* alone using *Amhr2*-Cre. These possibilities can be further exploited using uterine epithelial-specific Cre drivers in the future.

Our unbiased transcriptomic analysis identified enrichment of several signaling pathways in the uteri of *Ezh2 Amhr2*-Cre cKO mice. Although the potential significance of altered NK cell-mediated cytotoxicity remains unclear in premature *Ezh2 Amhr2*-Cre cKO mice, uNK cells are important during pregnancy establishment [51]. Reduced numbers of uNK cells were previously found in the decidua of *Ezh2 Pgr*-Cre cKO mice [17,19]. In the current study, we showed decreased mRNA expression of several NK cell-associated genes and reduced abundance of PRF1-postive cells in the decidua of *Ezh2 Amhr2*-Cre cKO mice. As uNK cells express PRF1 [54] and are the most dominant lymphocytes in the implantation site [55], these findings together with the histological observation using PAS staining suggest declined abundance of uNKs in the decidua of *Ezh2 Amhr2*-Cre cKO mice. Collectively, our results support an important role of EZH2 in maintaining decidual integrity. RNA-seq also revealed the enrichment of hedgehog signaling and RA signaling pathways in the uteri of *Ezh2 Amhr2*-Cre cKO mice. Hedgehog signaling is involved in the development of the uterus and vagina [56]. IHH is expressed in uterine and vaginal epithelia and activates hedgehog signaling in the stroma, where *Gli* genes are expressed [56]. The role of hedgehog signaling in uterine gland development is unclear. In the uterus of neonatal mice, hedgehog signaling regulates uterine epithelial cell proliferation [56], which plays a permissive role for uterine gland development [57,58]. However, expression of a dominant active SMO in the Mullerian duct results in reduced numbers of uterine glands [59]. Thus, normal hedgehog signaling is important for appropriate morphogenesis of uterine glands. 

Of particular interest is the finding of dysregulated RA signaling in the *Ezh2 Amhr2*-Cre cKO mouse model. RA plays fundamental roles in developmental events [60]. Dysregulation of RA signaling is associated with various pathological changes. RA signaling is known to regulate uterine stromal cell differentiation [61]. Recent evidence indicates that RA signaling is essential for endometrial function and fertility [62]. Disruption of RARs or RXRs leads to developmental abnormalities of the Mullerian duct [63]. RA and its receptors are present in the epithelium and stroma of the uterus and vagina [63]. Inhibition of RA signaling by feeding mice with vitamin A-deficient diets causes squamous metaplasia in the uterus [64], suggesting a role of RA signaling in uterine epithelial development. Evidence from other systems indicates that RA signaling links to epithelial duct or gland development [35,36]. It has also been reported that inhibition of RA in vivo results in excessive mammary ductal morphogenesis, which is reversible by RA treatment [36]. In contrast, RA signaling is important for salivary gland initiation [35]. These studies suggest a tissue-specific function of RA in glandular/epithelial development. 

A recent study using single cell RNA-sequencing has revealed the involvement of RA signaling in early uterine epithelial development in mice [65]. RA metabolism-related genes are expressed in uterine epithelial cells at various time stages, with a peak at PD14 [65]. It has been reported that the expression of *Aldh1a1*, a dehydrogenase facilitating the metabolism of retinol [66], is linked to epithelial progenitor cells within the developing uterine glands [65]. Inhibition of ALDH1 impairs uterine epithelial proliferation [65]. *Aldh1a1* was downregulated at PD10 in the uteri of *Ezh2 Amhr2*-Cre cKO mice, concomitant with impaired uterine gland formation. However, at the same developmental stage, mRNA levels for *Dhrs9*, *aldh3b2*, *Crabp1*, *Crabp2*, and *Rxrg* were upregulated in the uteri of *Ezh2 Amhr2*-Cre cKO mice. Due to the limitation of bulk RNA-seq, further studies using single-cell transcriptomics and epigenomics are necessary to define the cellular landscape of RA signaling and the gene regulatory network associated with uterine adenogenesis. It is important to assess the status of RA signaling activation/inhibition and its role in the epithelial versus stromal compartments during adenogenesis, as epithelial-stromal interactions play an important role in uterine development [67]. Studies using reporter mice expressing LacZ under the control of RA responsive element (RARE) (RARE-hsp68LacZ mice) [68] may be beneficial to assess the cell-type specific changes of RA signaling and its implication in uterine gland development. 

WNT signaling is instrumental for uterine gland development; and abnormal WNT signaling disrupts uterine gland formation and causes infertility in mice [28,57]. However, our RNA-seq and bioinformatics analysis did not identify the enrichment of WNT signaling. Upon examination of the expression profile of WNT-related genes, we found that the fold changes of many of WNT-related genes were below 2, the threshold of our bioinformatics analysis to filter out DE genes. It was interesting to note the increased expression of WNT inhibitors *Sfrp4* (Log2 FC = 0.45) and *Dkk2* (Log2 FC = 0.51) in *Ezh2 Amhr2*-Cre cKO mice (Ni N. and Li Q., unpublished observation). Single cell analysis in the future may help better define EZH2-directed signaling circuitry that controls uterine adenogenesis. 

Of note, uterine gland secretions are also important for pregnancy success in the human, and impaired development of uterine glands and/or their functions may be linked to pregnancy complications (e.g., miscarriage and preeclampsia) and pregnancy loss [69]. Due to the limitation to obtaining human specimens during pregnancy, our mouse model may be useful in defining mechanisms underlying uterine gland development and function. Further investigations are needed to determine whether and how histone modifications are involved in adenogenesis and the potential translational implication of our mouse model and findings in understanding uterine gland biology and pregnancy failure.

In summary, this study has identified a novel role of EZH2 in uterine gland development. Conditional deletion of *Ezh2* using *Amhr2*-Cre impairs uterine gland formation, accompanied by dysregulation of genes associated with critical developmental pathways (Supplementary Figure S9). The mouse model developed by this study may prove useful to understand epigenetic regulation of uterine development and function.

## 4. Materials and Methods

### 4.1. Ethics Statement

All animal protocols were approved by the Institutional Animal Care and Use Committee at Texas A&M University. Mice were maintained on a C57BL/6/129SvEv background and handled according to the guidelines of National Institute of Health. Mice were housed under a 12 h light/12 h dark cycle in the Laboratory Animal Resources and Research facility located in Texas A&M University, with free access to water and food during the entire experimental period. Animal care was provided by the research staff under the comparative medicine program and experienced research personnel. All necessary procedures were taken to minimize the discomfort and pain.

### 4.2. Animal Models, Treatments, and Tissue Collections

*Ezh2^flox/flox^* mice were purchased from the Jackson Laboratory (Stock no. 022616) and crossed with *Amhr2*-Cre mice [31] to produce *Ezh2 Amhr2*-Cre conditional knockout (cKO) mice. Genotyping of *Ezh2* and *Amhr2*-Cre and analysis of recombination of the *Ezh2* conditional allele were performed using genomic PCR as described elsewhere [19,70]. The day of copulation plug was defined as embryonic day (E) 0.5. Implantation sites in *Ezh2 Amhr2*-Cre cKO female mice and controls at E4.5 were visualized by tail vein injection of 1% Chicago Sky Blue dye (MilliporeSigma, Burlington, MA, USA). Uterine/ovarian tissue samples and implantation sites were collected from *Ezh2 Amhr2*-Cre cKO mice and age-matched controls. Uterine tissues for histological and immunohistochemical analyses were fixed with 10% neutral buffered formalin (NBF) and embedded in paraffin, whereas those used for RNA isolation were processed using QIAGEN RNA isolation kit according to the manufacturer’s instructions. Positive controls for TRP63 and KRT14 immunostaining were uterine tissues collected from 8-month-old *Pgr*-Cre mice in a previous study [19]. Fertility test was performed by continuously breeding of *Ezh2 Amhr2*-Cre cKO females and controls with known fertile males for a period of 3 months. The number of pups/litter and litter/month were calculated.

### 4.3. Histological Analysis and Immunostaining

Hematoxylin and eosin (H.E.) staining, Periodic Acid Schiff (PAS) staining, and immunohistochemistry were performed using paraffin embedded sections [71]. Primary antibodies with appropriate dilutions were used to detect target proteins (Table 1). An Avidin/Biotin Complex (ABC) protocol was used to amplify the immunoreactive signals, with the sequential addition of secondary biotinylated anti-rabbit antibody (BA-1000, Vector laboratories) or anti-rat (BA-9400) antibody and ABC reagents (Vector Laboratories, Burlingame, CA, USA). The NovaRED^™^ Peroxidase Substrate Kit (Vector Laboratories) was utilized to develop signals for the targets. Quantitative analysis of uterine glands was carried out by counting the number of forkhead box A2 (FOXA2)-positive glands on the cross sections of control and *Ezh2 Amhr2*-Cre cKO mice. At least 11 sections from each uterine sample were analyzed. To determine differences in the abundance of perforin 1 (PRF1)-expressing cells between control and *Ezh2 Amhr2*-Cre cKO mice at E6.5 and E7.5, immunostaining was performed using anti-PRF1 antibody and the number of PRF1-positive cells quantified using the Qupath software [72]. 

### 4.4. Quantitative Reverse Transcription (qRT)-PCR

RNA isolation and qRT-PCR were conducted as described [71]. Superscript III-based RT was performed using approximately 500 ng of total RNA per reaction. qRT-PCR was conducted using cDNA, iTaq Universal SYBR Green Supermix (Bio-Rad, Hercules, CA, USA) or Taqman PCR Master Mix (ThermoFisher Scientific, Waltham, MA, USA), and oligo primers for SYBR green analysis or TaqMan probes for TaqMan gene expression analysis. Oligo primers were synthesized by ThermoFisher Scientific for *Ezh2* [19], *Foxa2* [19], killer cell lectin-like receptor subfamily G, member 1 (*Klrg1*) [19], *Prf1* [19], bone morphogenetic protein 2 (*Bmp2*) [73], follistatin (*Fst*) [73], heart and neural crest derivatives expressed 2 (*Hand2*) [74], fibroblast growth factor 9 (*Fgf9*) [75], fibroblast growth factor 18 (*Fgf18*) [75], GLI-Kruppel family member GLI1 (*Gli1*) [71], aldehyde dehydrogenase 3 family, member B2 (*Aldh3b2*) [76], interleukin 11 receptor, alpha chain 1 (*Il11ra1*) [77], and others [78,79] (Table 2). Gene expression was determined using ribosomal protein L19 (*Rpl19*) as an internal control [19]. TaqMan probes for WNT family member 5A (*Wnt5a*) (Mm00437347), chemokine (C-X-C motif) ligand 15 (*Cxcl15*) (Mm00441263), WAP four-disulfide core domain 3 (*Wfdc3*) (Mm01243777), prolactin family 8, subfamily a, member 2 (*Prl8a2*) (Mm01135453), and *Rpl19* (Mm02601633) were purchased from ThermoFisher Scientific. Assays were performed using CFX Connect (Bio-Rad), with at least 3 biological replicates and 2 technical replicates for each sample. 

### 4.5. Whole-Mount Immunofluorescent Staining and 3D Imaging of the Uterus

Uterine horns were collected from control and *Ezh2 Amhr2*-Cre cKO mice at the age of 1 month. The specimens were fixed in 10% NBF at room temperature for 16 h and washed with phosphate-buffered saline (PBS). Uterine horns were cleared and labeled using a modified iDISCO + procedure [80,81]. Briefly, samples were dehydrated in methanol/H_2_O series for 6 h, incubated in 66% dichloromethane (DCM)/33% methanol overnight, washed with 100% methanol twice for 30 min, and bleached in 5% hydrogen peroxide in methanol overnight at 4 °C. Samples were then rehydrated, permeabilized, blocked and whole-mount immunofluorescent labeling of FOXA2 was performed using anti-FOXA2 antibody (Table 1) at 37 °C for 2 days. Then, samples were washed with PTwH buffer containing 0.2% Tween-20 and 10 µg/mL Heparin in 1X PBS for 5 times, with 30 min each for the first 4 washes and overnight for the 5th and final wash. Then, the uterine horns were incubated with an Alexa Fluor 594-conjugated secondary antibody (1:250, ThermoFisher Scientific) at 37 °C for 2 days. The samples were washed, mounted in 2% agarose, and dehydrated again in methanol/H_2_O series. Then, they were incubated in 66% dichloromethane (DCM)/33% methanol for 3 h, washed with 100% DCM, and transferred to Ethyl Cinnamate for refractive index matching. A Zeiss Lightsheet Z.1 Light Sheet Fluorescence microscope was used to capture images from the whole-mount uteri. Images were processed using Imaris software (Bitplane, Zürich, Switzerland). 

### 4.6. Hormone Assay

Sera were collected from control and *Ezh2 Amhr2*-Cre cKO mice at 3 months of age and E8.5. Serum levels of E2, progesterone (P4), follicle-stimulating hormone (FSH), and luteinizing hormone (LH) were measured using the Ligand Assay and Analysis Core, Center for Research in Reproduction, University of Virginia. Assay information is available at https://med.virginia.edu/research-in-reproduction/ligand-assay-analysis-core/assay-methods/ (accessed on 3 March 2022).

### 4.7. RNA-Sequencing and Bioinformatics Analysis

RNA-sequencing (RNA-seq) was performed using Illumina NavaSeq 6000 at Texas A&M Institute for Genome Sciences and Society. Total RNA was extracted from the uteri of *Ezh2 Amhr2*-Cre cKO mice and controls at PD10 (*n* = 4) using RNA mini kit (QIAGEN, Germantown, MD, USA). The quality of RNA was checked by Agilent Bioanalyzer. Bioinformatics analysis of RNA-seq data was performed using the Galaxy public server (usegalaxy.org). The data were deposited to Gene Expression Omnibus (GEO) (GSE201973). In brief, data were imported and analyzed using the following pipeline. First, Trimmomatic was used to trim poor quality bases and remove low quality reads [82]. Quality check was performed using FastQC (http://www.bioinformatics.babraham.ac.uk/projects/fastqc/) (accessed on 22 April 2022). Next, sample reads were aligned to the mouse genome (GRCm38_mouse, also known as mm10) using HISAT2 algorithm [83] and counted using FeatureCounts [84]. Finally, differential gene expression was analyzed using EdgeR [85]. Genes with a count-per-million (CPM) greater than 0.2 in 4 samples were kept for further analysis. Adjusted *p*-values were generated using Benjamini and Hochberg (1995) method [86]. A trimmed mean of M values (TMM) method was applied to estimate relevant scaling factors to account for the variation of library size. The robust setting was turned on (i.e., robust = TRUE) to protect against possible outlier genes. Genes with fold change greater than 2 or less than −2 and adjusted *p*-value less than 0.05 were depicted as differentially expressed (DE) genes. Heatmap2 was used to generate the heatmap for DE genes. Volcano plot was created using volcano plot tool in the Galaxy database, with selection of adjusted *p* values instead of raw *p* values for y axis plotting. g:Profiler [87], a web server, was used to identify enriched biological terms from the DE gene list using KEGG and REAC databases.

### 4.8. Statistical Analysis

Differences between two means for qRT-PCR, uterine gland quantification, PRF1-positive cell quantification, number of blastocysts, and hormone levels were determined by two-tailed *t*-test (unpaired). Data are presented as mean ± s.e.m. Statistical significance was reported when a *p* value is less than 0.05, with results marked as * *p* < 0.05, ** *p* < 0.01 and *** *p* < 0.001.

## Figures and Tables

**Table 1:** Primary antibody information.

Name	Catalog No.	Manufacturer	Host	Immunostaining
EZH2	5246	Cell signaling, Danvers, MA	Rabbit	1:200
FOXA2	Ab108422	Abcam, Waltham, MA USA	Rabbit	1:250 (IHC) 1:400 (WMS)
FOXA2	8186	Cell signaling	Rabbit	1:200
ACTA2	19245	Cell signaling	Rabbit	1:500
KRT14	PA5-16722	Thermo Fisher	Rabbit	1:400
TRP63	13109	Cell signaling	Rabbit	1:200
CRABP1	13163	Cell signaling	Rabbit	1:400
PRF1	31647	Cell signaling	Rabbit	1:200
VIM	5741	Cell signaling	Rabbit	1:200
KRT8	TROMA-I	DSHB, Iowa City, USA	Rat	1:200
PTGS2	12282	Cell signaling	Rabbit	1:600
Desmin	Ab32362	Abcam	Rabbit	1:500

IHC, immunohistochemistry; WMS, whole-mount staining.

**Table 2:** qRT-PCR primers.

Gene	F/R	Sequence (5′-3′)	Reference
*Hoxa5*	F	CTCATTTTGCGGTCGCTATCC	PrimerBank ID: 6754232a1
	R	ATCCATGCCATTGTAGCCGTA	
*Upk1a*	F	GGGCAACATCATTATTTTGCTGT	PrimerBank ID: 12835021a1
	R	CGTGAGGATCATGTACCGACG	
*Krt15*	F	AGCTATTGCAGAGAAAAACCGT	PrimerBank ID: 6680602a1
	R	GGTCCGTCTCAGGTCTGTG	
*Aqp5*	F	AGAAGGAGGTGTGTTCAGTTGC	PrimerBank ID: 6857757a1
	R	GCCAGAGTAATGGCCGGAT	
*Ihh*	F	CTCTTGCCTACAAGCAGTTCA	PrimerBank ID: 14149643a1
	R	CCGTGTTCTCCTCGTCCTT	
*Hhip*	F	TGAAGATGCTCTCGTTTAAGCTG	PrimerBank ID: 34328503a1
	R	CCACCACACAGGATCTCTCC	
*Ptch1*	F	AAAGAACTGCGGCAAGTTTTTG	PrimerBank ID: 6679519a1
	R	CTTCTCCTATCTTCTGACGGGT	
*Ptch2*	F	CTCCGCACCTCATATCCTAGC	PrimerBank ID: 6679517a1
	R	TCCCAGGAAGAGCACTTTGC	
*Spp1*	F	AGCAAGAAACTCTTCCAAGCAA	PrimerBank ID: 6678113a1
	R	GTGAGATTCGTCAGATTCATCCG	
*Rdh1*	F	GTCATGGGCCGAATGTCTTTC	PrimerBank ID: 20147789a1
	R	CACAAGTCTTGAAGCCTCCAG	
*Dhrs9*	F	ATGCTGTTTTGGTTGTTGGCT	PrimerBank ID: 30425272a1
	R	GTTCTGGCTGCTAAGTTTCCA	
*Crabp1*	F	CAGCAGCGAGAATTTCGACGA	PrimerBank ID: 7304975a1
	R	CGCACAGTAGTGGATGTCTTGA	
*Crabp2*	F	ATGCCTAACTTTTCTGGCAACT	PrimerBank ID: 33469075a1
	R	GCACAGTGGTGGAGGTTTTGA	
*Rxrg*	F	CATGAGCCCTTCAGTAGCCTT	PrimerBank ID: 6677829a1
	R	CGGAGAGCCAAGAGCATTGAG	
*Stra6*	F	CTGGTACATCGAGGAACCTCT	PrimerBank ID: 6678171a1
	R	CCAGGAACGACAGTGAAGCC	
*Cyp26a1*	F	AAGCTCTGGGACCTGTACTGT	PrimerBank ID: 6681101a1
	R	CTCCGCTGAAGCACCATCT	
*Aldh1a1*	F	ATACTTGTCGGATTTAGGAGGCT	PrimerBank ID: 7304881a1
	R	GGGCCTATCTTCCAAATGAACA	
*Aldh1a3*	F	GGGTCACACTGGAGCTAGGA	PrimerBank ID: 31542123a1
	R	CTGGCCTCTTCTTGGCGAA	

F, forward primer; R, reverse primer.

## Data Availability

Data are included in the main article and Supplementary Figures.

## Supplementary Materials

The supporting information can be downloaded at: https://www.mdpi.com/article/10.3390/ijms232415665/s1.
