# Supplementary material for: Role of EZH2 in Uterine Gland Development
Source: Int J Mol Sci. 2022 Dec 10;23(24):15665. doi: 10.3390/ijms232415665 (PMC9779349; doi:10.3390/ijms232415665)
Supplement: Supplementary file 1 [file ijms-23-15665-s001.zip › ijms-1897944-supplementary.pdf]

Role of EZH2 in Uterine Gland Development

Nan Ni[1], Frank L. Jalufka[2], Xin Fang[1], Dylan A. McCreedy[2,3,4], Qinglei Li[1,*]

[1]Department of Veterinary Integrative Biosciences, Texas A&M University, College Station, TX 77843, USA

[2]Department of Biology, Texas A&M University, College Station, TX 77843, USA

[3]Department of Biomedical Engineering, Texas A&M University, College Station, TX 77843, USA

[4]Institute for Neuroscience, Texas A&M University, College Station, TX 77843, USA

*Corresponding author

Supplementary Materials

Figure S1. Generation of *Ezh2 Amhr2*-Cre cKO mice.

Figure S2. Immunohistochemical analysis of uterine development in *Ezh2* conditional knockout mice.

Figure S3. Histological analysis of uteri from *Ezh2 Amhr2*-Cre cKO mice.

Figure S4. qRT-PCR analysis of uterine gland- and adenogenesis-associate genes using uteri from control and *Ezh2 Amhr2*-Cre cKO mice at PD10.

Figure S5. Ovarian histology and function of *Ezh2 Amhr2*-Cre cKO mice.

Figure S6. Conditional deletion of *Ezh2* using *Amhr2*-Cre does not cause epithelial stratification.

Figure S7. Reduced numbers of PRF1-positive cells in *Ezh2 Amhr2*-Cre cKO mice at E6.5.

Figure S8. PAS staining of uNK cells.

Figure S9. A graphical summary.

Table S1. Differentially expressed genes in the uteri of *Ezh2 Amhr2*-Cre cKO mice versus controls at PD10.

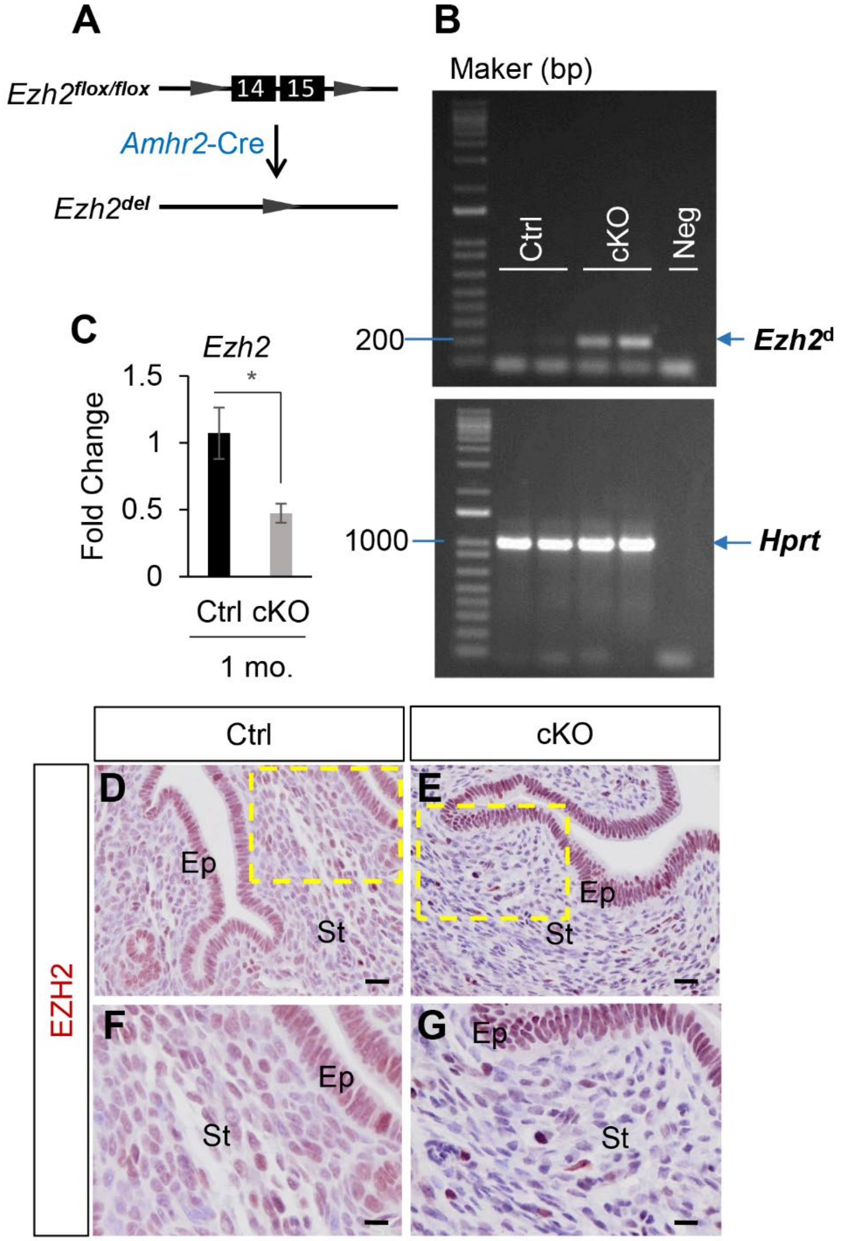

Figure S1. Generation of *Ezh2 Amhr2*-Cre cKO mice. (**A**) Cre-loxP strategy to generate conditional deletion of *Ezh2* using *Amhr2*-Cre. (**B**) Recombination PCR. Note that the recombined band of *Ezh2* was only detectable in the uteri of *Ezh2 Amhr2*-Cre cKO mice but not controls. Neg, negative control. (**C**) qRT-PCR analysis of *Ezh2* mRNA levels using uteri from 1-month-old control and *Ezh2 Amhr2*-Cre cKO mice. n=5. *Rpl19* was included as an internal control. Data are mean ± s.e.m, *p<0.05. (**D-G**) Immunostaining of EZH2 using uteri of 1-month-old control and *Ezh2 Amhr2*-Cre cKO mice. Panels (F) and (G) are high power images of the boxed regions of panels (D) and (E), respectively. At least three animals for each genotype were examined. St, stroma. Ep, epithelium. Scale bar = 10 μm (F and G) and 20 μm (D and E).

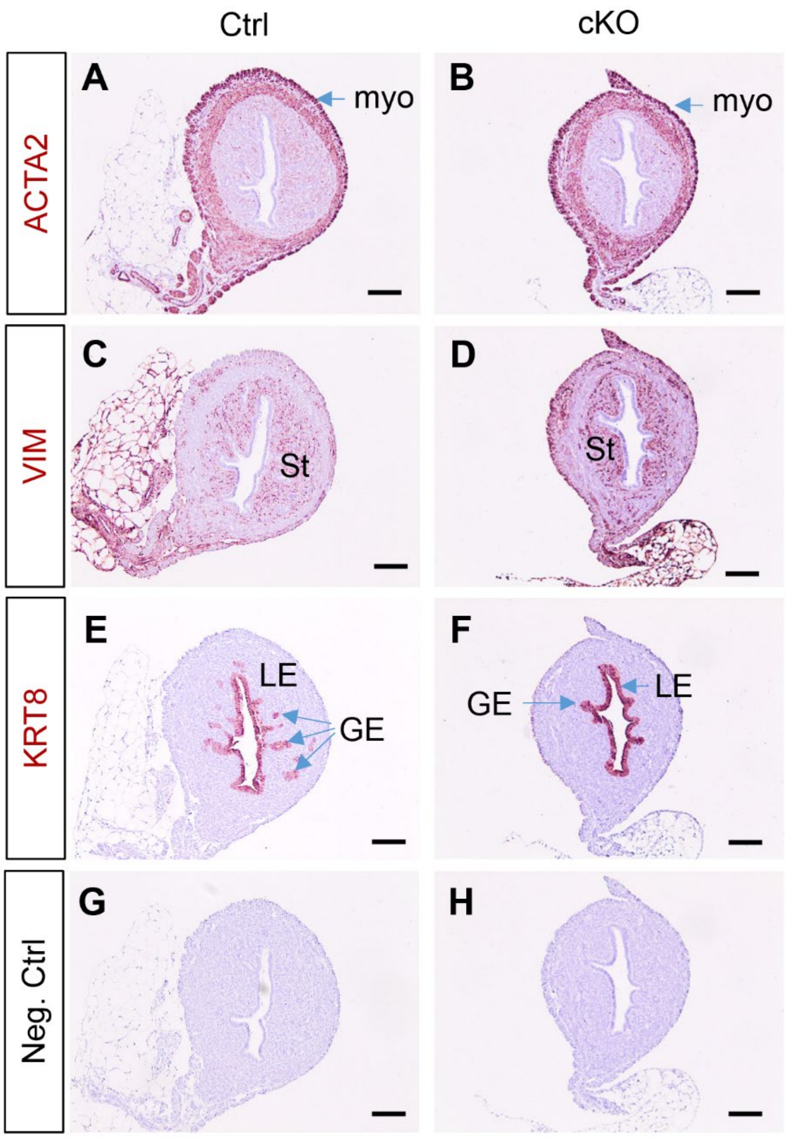

Figure S2. Immunohistochemical analysis of uterine development in *Ezh2* conditional knockout mice. (**A-F**) Immunostaining of ACTA2, VIM, KRT8 using uteri from control and *Ezh2 Amhr2*-Cre cKO mice at PD15. At least three animals for each genotype were examined. (**G** and **H**) Negative controls using rabbit IgG. Scale bar=100 μm (A-H).

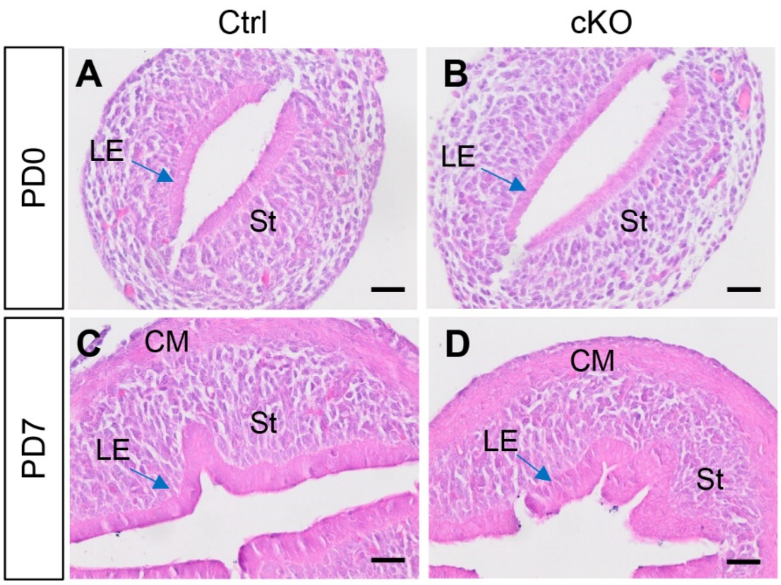

Figure S3. Histological analysis of uteri from *Ezh2 Amhr2*-Cre cKO mice. (**A-D**) H.E. staining of uteri from control and *Ezh2 Amhr2*-Cre cKO mice at PD0 and PD7. At least three animals for each genotype were examined. LE, luminal epithelium. St, stroma. CM, circular muscle layer. Scale bar=25 μm (A-D).

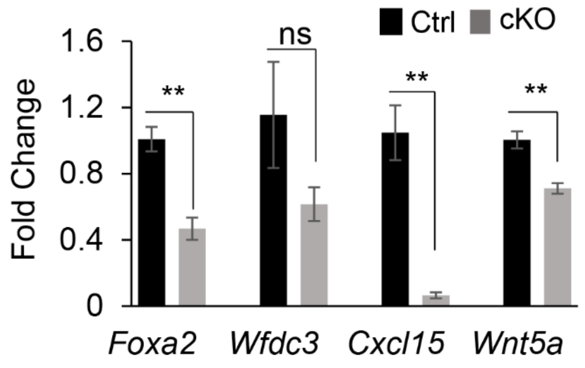

Figure S4. qRT-PCR analysis of uterine gland- and adenogenesis-associate genes using uteri from control and *Ezh2 Amhr2*-Cre cKO mice at PD10. n=4. Data are mean ± s.e.m, ***p*<0.01. ns, not significant.

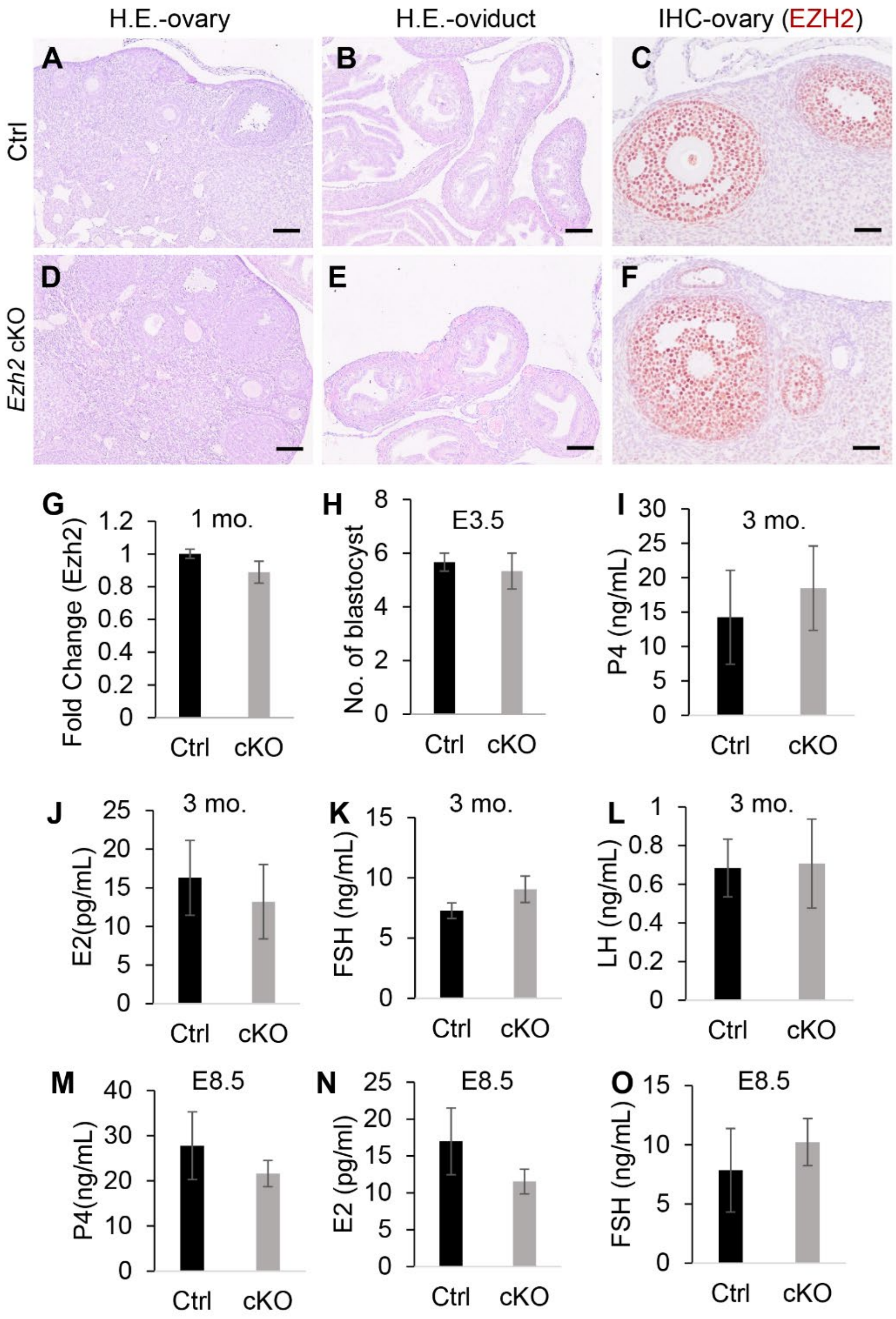

Figure S5. Ovarian histology and function of *Ezh2 Amhr2*-Cre cKO mice. (**A-F**) Histological analysis and EZH2 immunostaining using 2-month-old control and *Ezh2 Amhr2*-Cre cKO mice. IHC, immunohistochemistry. Scale bar=50 μm (C and F) and 100 μm (A, B, D, and E). At least three animals for each genotype were examined. (**G**) qRT-PCR analysis of *Ezh2* mRNA levels using ovaries from 1-month-old control and *Ezh2 Amhr2*-Cre cKO mice. n=4. Data are mean ± s.e.m, (**H**) Number of blastocysts recovered from the uteri of control and *Ezh2 Amhr2*-Cre cKO mice at E3.5. n=3. (**I-O**) Hormone levels in the serum of control and *Ezh2 Amhr2*-Cre cKO mice at 3 months of age and/or E8.5. n=3-5. Data are mean ± s.e.m.

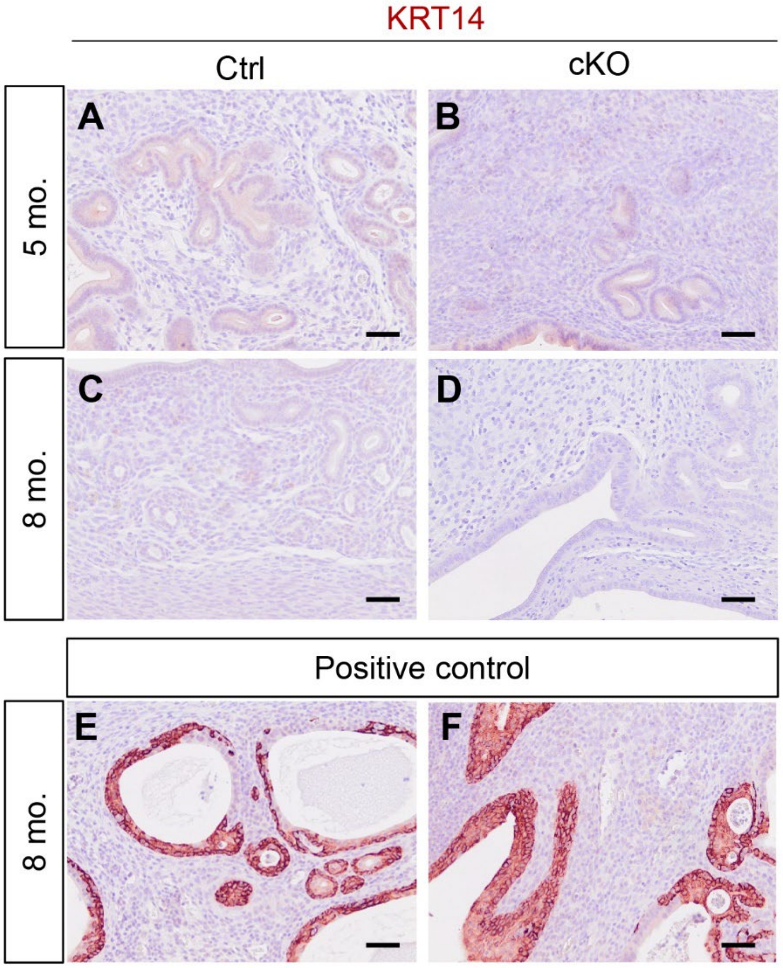

Figure S6. Conditional deletion of *Ezh2* using *Amhr2*-Cre does not cause epithelial stratification. (**A-D**) Immunostaining of KRT14 using uteri from control and *Ezh2 Amhr2*-Cre cKO mice at 5 and 8 months of age. At least three animals for each genotype were examined. (**E** and **F**) Immunostaining of KRT14 using uteri from an 8-month-old *Ezh2 Pgr*-Cre cKO mouse as the positive control. Panels (E) and (F) represent different fields of the cross section. Scale bar=50 μm (A-F).

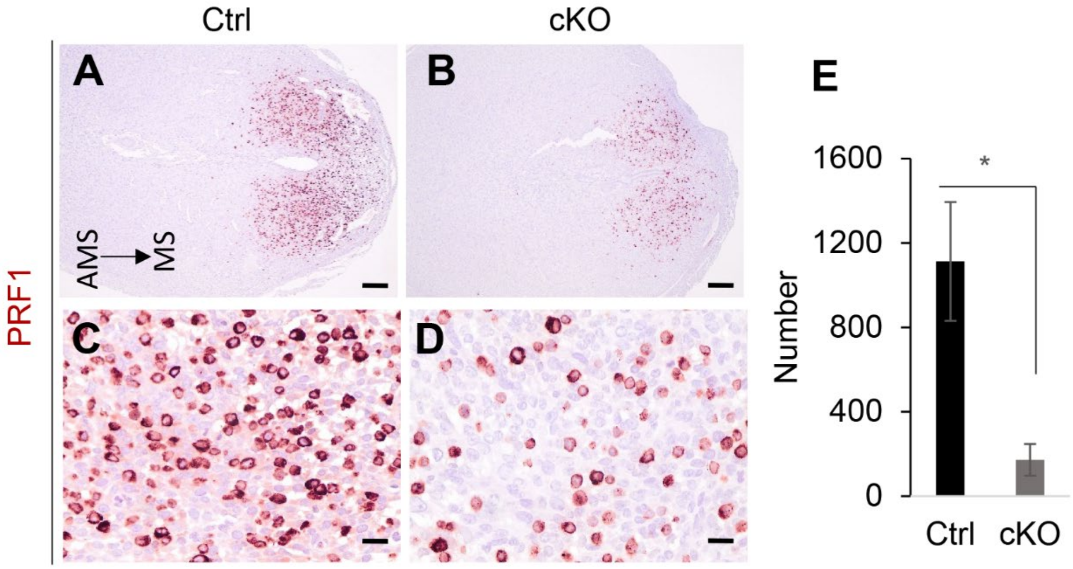

Figure S7. Reduced numbers of PRF1-positive cells in *Ezh2 Amhr2*-Cre cKO mice at E6.5.(**A-D**) Immunostaining of PRF1 using uteri from control and *Ezh2 Amhr2*-Cre cKO mice at E6.5. At least three animals for each genotype were examined. Panels (C) and (D) are higher power images for panels (A) and (B), respectively. MS, mesometrial side. AMS, antimesometrial side. Scale bar = 40 µm (C and D) and 200 µm (A and B). (**E**) Qualification of the number of PRF1-positive cells in control and *Ezh2 Amhr2*-Cre cKO mice E6.5. n=3. Data are mean ± s.e.m. *p<0.05.

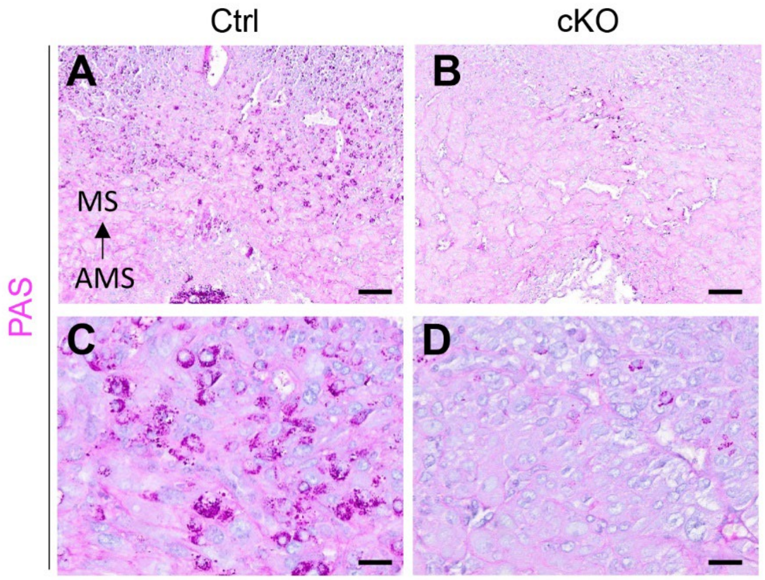

Figure S8. PAS staining of uNK cells. (**A-D**) PAS staining using uteri from control and *Ezh2 Amhr2*-Cre cKO mice at E7.5. At least three animals for each genotype were examined. Panels (C) and (D) are higher power images for panels (A) and (B), respectively. MS, mesometrial side. AMS, antimesometrial side. Scale bar = 25 μm (C and D) and 100 μm (A and B).

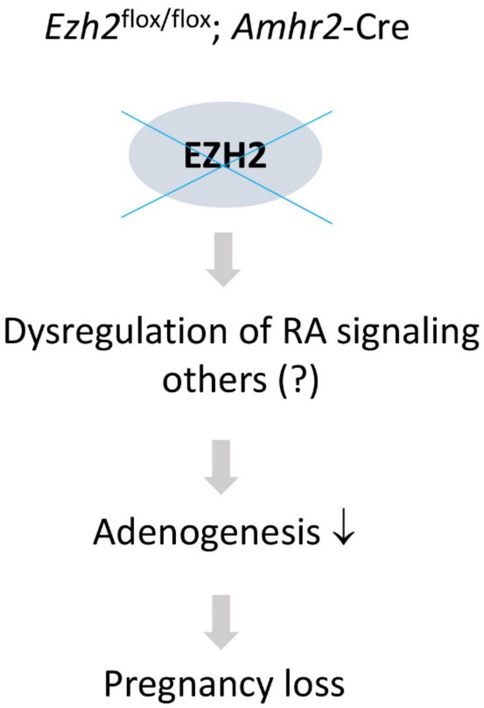

Figure S9. A graphical summary.

Table S1. Differentially expressed genes in the uteri of *Ezh2 Amhr2*-Cre cKO mice versus controls at PD10

SYMBOL	logFC	logCPM	F	PValue	FDR
Myh3	4.084410852	-0.427874733	39.21844865	4.66E-05	0.003882812
a	3.635512129	1.718317727	268.2509477	7.01E-10	5.84E-06
Fndc3c1	3.221869093	1.473370855	188.4191887	5.83E-09	1.39E-05
Hoxd13	3.207972923	0.648094781	93.23586383	3.44E-07	0.000163561
Retnla	3.083915501	-0.077922766	27.19447134	0.000284309	0.013119186
AI606473	2.938179223	-1.609483258	26.88133663	0.000193008	0.010304876
Igf2bp1	2.913814703	1.649762299	194.1149698	4.89E-09	1.36E-05
Usp44	2.600131962	-1.851882797	31.5776308	9.31E-05	0.006157233
Klb	2.585024537	-0.693712692	19.62038265	0.00084561	0.025564743
Lamp5	2.56845684	-1.72686635	32.82708357	7.77E-05	0.005439137
Mcpt2	2.484975273	-1.008927421	29.90045629	0.000119616	0.007219451
Robo3	2.478208061	-0.609275655	56.39265639	5.28E-06	0.000907092
B3galt2	2.430664013	0.873026967	19.76944299	0.001257723	0.032132266
Mab21l1	2.308422879	-0.18956687	47.11813791	1.33E-05	0.001666486
Grem1	2.293964036	1.626688945	103.4661058	1.91E-07	0.000117829
Lhx8	2.247975816	-0.10497445	43.0298378	2.10E-05	0.002314183
Adig	2.24617137	-0.243829732	22.60277425	0.000453188	0.017679655
Gm38427	2.224154519	-1.522346341	24.72085654	0.000278408	0.012942696
Mslnl	2.151453443	-1.468734279	19.71834222	0.000713745	0.023060807
Drd1	2.145852493	-1.57453922	16.22049717	0.001518706	0.035736036
Hpca	2.133407306	-1.40305531	16.86771914	0.001311264	0.032857189
Lgals12	2.091268328	1.059896794	40.17098088	3.65E-05	0.003402877
Eef1a2	2.090201167	-2.037679119	16.57948355	0.00139929	0.034024053
Col6a5	1.998901792	4.166614552	173.7845323	2.19E-08	3.04E-05
Mir681	1.971352205	-1.687351038	17.09518102	0.001246322	0.032020933
Cpn2	1.868255703	-1.041177408	17.15757057	0.001229168	0.031843673
Abcb11	1.858866046	-1.852649341	18.2699279	0.000964955	0.027628836
Six1	1.83066887	1.195912414	55.55801041	5.71E-06	0.000960839
Slc26a4	1.827434677	1.449649411	24.12032755	0.000488769	0.018234704
Lrrtm3	1.802212419	-0.109206567	22.68178078	0.000401736	0.016288031
Actc1	1.800742081	0.521849137	23.76794558	0.000368845	0.015437753
Pcsk1n	1.78537284	-1.03776586	29.37041827	0.000129759	0.007591245
Acan	1.750340054	-1.026701926	16.82654609	0.001323435	0.033052142
Mc2r	1.737534638	-1.003869959	14.55129	0.002256225	0.045722872
Dhrs9	1.735590075	1.485109943	42.98428098	2.23E-05	0.002393886
Olig3	1.713023459	-1.519098566	14.70434154	0.002173447	0.044668028
Serpinb11	1.695161995	-0.740943853	14.20285325	0.002458748	0.047675573
Hoxaas3	1.691066628	-1.266690464	18.71437328	0.000878289	0.02583397
Nxph1	1.683504492	-0.06896344	17.21501269	0.001243655	0.032020933
Zfhx4	1.660744056	3.463497025	106.0287615	2.12E-07	0.000126295
Rbfox3	1.658910428	0.484732299	53.88316894	6.70E-06	0.001042662
Gm10419	1.643767182	-0.945417653	18.70874392	0.000879329	0.02583397
Adam33	1.640938537	1.667430799	22.819792	0.000593095	0.020669004
Pparg	1.631049242	1.280550248	16.62062581	0.001985105	0.041805154
Slc7a10	1.60427873	1.60686362	37.43908404	4.54E-05	0.003871994
Hoxa2	1.595098297	0.30059504	40.08859214	2.98E-05	0.002941106

Tcf24	1.587986765	-1.079851299	18.15397506	0.000989174	0.027855688
Hoxa6	1.577288953	-0.40402004	25.77853137	0.000232072	0.011437441
Hoxa5	1.528740501	1.669664182	51.01253955	8.89E-06	0.001213622
Zim1	1.526558607	2.985311993	87.16669465	5.01E-07	0.00021947
Lmx1a	1.511384219	-0.894191856	16.474981	0.001432888	0.034471509
Npb	1.504377245	-1.789588164	15.67867195	0.001722139	0.038714419
Kcnt1	1.494570184	0.609962614	32.95596105	7.63E-05	0.005412044
Miat	1.47615373	1.076062967	26.70217699	0.000201339	0.010580142
Agt	1.457869475	0.150617464	20.49138353	0.000611183	0.020865986
Hoxd12	1.455514543	0.05630062	21.62960954	0.000489714	0.018234704
Trim63	1.45201534	-0.798496255	16.47476404	0.001432959	0.034471509
Slc36a2	1.41923055	1.072651409	20.16516608	0.000730428	0.023398982
Klf14	1.408124101	1.663731792	61.73421286	3.28E-06	0.00067244
Kcnq2	1.399025074	-0.280892787	18.1773146	0.000984243	0.027789008
Slc10a4	1.379734475	0.589270425	29.8268091	0.000120969	0.00727474
Nr2f1	1.376430191	2.344575809	57.34523233	4.84E-06	0.000857342
Cxcl5	1.375344868	1.596620373	46.47687102	1.43E-05	0.001712704
Crabp1	1.359758319	5.914118978	24.92811448	0.001339754	0.033063151
Adrb3	1.337126507	0.938430176	44.39414861	1.80E-05	0.00203957
Hoxa3	1.336611544	2.358705125	107.1074286	1.57E-07	0.000104546
Il1r2	1.33224522	0.847644027	44.47480297	1.78E-05	0.00203957
Synpo2l	1.319497595	-0.517361165	16.59695241	0.001393763	0.034024053
Foxp2	1.310147178	3.285489006	139.0786465	3.48E-08	4.15E-05
Aldh3b2	1.305548604	0.310565715	20.71970133	0.00058424	0.020403082
Cdkn2a	1.298415159	-1.156004067	14.09981262	0.002522641	0.048414116
Hoxc9	1.287095979	-0.449808547	15.30159333	0.001882494	0.040766201
1700028K03Rik	1.286930423	-0.755119505	14.99728757	0.002024658	0.042286147
Foxd3	1.272844947	-1.13129635	15.49759547	0.001797072	0.040014646
Hoxc10	1.255826724	2.378920136	72.27059039	1.40E-06	0.000377374
Acvr1c	1.247551358	1.577235755	30.85594714	0.000103564	0.006528579
Erich3	1.239454415	1.864865318	44.09874819	1.86E-05	0.002080563
Cyp46a1	1.226659604	1.468139817	25.48266294	0.000244066	0.011922721
Hoxc5	1.210879268	-0.097190035	17.65067653	0.001102832	0.029678468
Bhlhe22	1.208293737	1.670713661	55.34978202	5.82E-06	0.000970062
Mug1	1.206507179	0.806371361	23.22064301	0.00036386	0.015267437
Crlf1	1.179301189	-0.193780913	16.51981417	0.001418361	0.034291799
Nnmt	1.176702571	0.892876507	18.474771	0.000923832	0.02667105
Catsperz	1.175816693	-0.150478203	18.0397517	0.001013725	0.027961086
Alx1	1.172814445	0.396964192	18.02003294	0.001018035	0.028030452
Susd5	1.152757195	0.502034761	22.0770532	0.000449815	0.017679655
A930013F10Rik	1.130967334	0.528306728	15.58443775	0.001760672	0.039421057
A830082K12Rik	1.128296468	0.907637305	33.0533223	7.53E-05	0.005412044
Cyp2f2	1.12352477	0.964916889	17.03378325	0.001263476	0.032181922
Arhgap40	1.116407865	1.589541622	17.71306973	0.001165154	0.030808143
Lmo3	1.109499758	2.290502529	52.40487484	7.74E-06	0.001120565
Prkcg	1.107199479	1.649252292	31.76779678	9.05E-05	0.00605652
Fabp7	1.086182875	0.472924614	16.80938828	0.001328546	0.033063151

Malat1	1.086081352	7.13007477	29.77603595	0.000522654	0.018968115
Hsf4	1.081256485	-0.258114579	14.19309263	0.002464719	0.047685589
Dlk1	1.076097163	7.719685347	129.6551896	8.02E-08	6.68E-05
Espn	1.070074729	2.635930989	56.42787404	5.26E-06	0.000907092
Tg	1.055451861	0.25003016	14.97828545	0.002033942	0.042404771
Cd300lg	1.053284272	2.244205905	35.18096466	5.61E-05	0.004363858
Zfp750	1.052161385	1.662047191	20.20617451	0.000646892	0.021527924
Gdf5	1.047979199	2.154390489	28.53300041	0.00014789	0.008507554
Arpp21	1.046749697	0.859573989	18.22352068	0.000974564	0.027703567
Prlr	1.04082467	3.786818187	30.42432772	0.000189635	0.010190153
Shisa6	1.029657278	-0.063341031	14.4386344	0.002319504	0.046654942
Lrrc29	1.025574388	1.292529406	19.22239919	0.00079008	0.024561816
Cdh19	1.024549402	0.162474637	14.42304404	0.002328422	0.046654942
Slc14a1	1.009006777	6.798662331	59.34565639	1.39E-05	0.001705991
Pcolce2	1.001722915	2.543503252	62.67361038	3.02E-06	0.000652565
Frmpd2	-1.002788987	0.348043571	19.68564319	0.000718506	0.023150629
Gimap3	-1.014451208	0.831996065	15.10249232	0.001974141	0.04170645
D430041D05Rik	-1.016426817	3.09905427	70.44052912	1.61E-06	0.000407456
Nxnl2	-1.019152958	0.725126437	23.43730449	0.000349811	0.014925536
P2ry14	-1.023622828	1.749203882	21.45598467	0.000506292	0.01849519
Ptch1	-1.025648403	5.722741655	91.35604329	7.02E-07	0.000248971
Prkg2	-1.043349066	2.285342669	56.90626873	5.04E-06	0.000883219
Scube1	-1.049902297	5.906390153	203.2672963	3.71E-09	1.36E-05
D630033O11Rik	-1.055439771	1.042474032	21.94853018	0.000460876	0.017854117
Bdkrb2	-1.059965173	2.789545547	61.97550043	3.21E-06	0.00067244
Ndp	-1.077717677	4.922405691	124.6371759	6.58E-08	6.28E-05
Cd83	-1.082588811	5.368503574	124.3523251	7.16E-08	6.28E-05
Atp6v0d2	-1.089979883	0.281021482	21.12122797	0.00054013	0.01939114
Aff2	-1.151951985	3.321028332	61.67891506	3.29E-06	0.00067244
Inmt	-1.168512289	0.501841812	13.90685506	0.00264756	0.049609736
Ptpro	-1.174509366	2.835639468	72.5597303	1.37E-06	0.000377374
Ascl2	-1.175063759	-0.822378795	14.26132188	0.002423332	0.047103693
Gna14	-1.183426114	3.831887731	128.3454828	5.55E-08	5.86E-05
Fam189a1	-1.188296741	0.520205239	35.31486495	5.51E-05	0.004346625
Slc5a11	-1.189428686	2.648166647	33.44734066	8.30E-05	0.005763215
Pthlh	-1.194153217	2.489191118	37.2390578	4.34E-05	0.003828223
Plac8	-1.204271809	6.248509674	119.4905667	2.47E-07	0.000128768
Gstm6	-1.231701147	-0.105526973	15.48229354	0.001803577	0.040027892
Batf3	-1.231728204	-0.493278306	18.84236872	0.000855031	0.025709592
Plcxd3	-1.232770939	-0.754459866	15.80018527	0.00167389	0.038075032
F830016B08Rik	-1.247205018	1.308577019	24.03609764	0.000314141	0.013954583
Cd226	-1.256709438	-0.680467277	21.04239549	0.000548477	0.019564287
Stra6	-1.268145913	6.005993393	138.3644109	8.86E-08	7.03E-05
Agtr1b	-1.285993879	-0.152446235	22.4697588	0.00041788	0.016773616
Klrb1c	-1.289996239	0.519124724	22.44233241	0.000420023	0.016819103
Synpr	-1.290543515	1.171452504	19.25391632	0.00083691	0.025401552
Eda2r	-1.2912698	5.043635041	63.42140712	1.10E-05	0.00143634

Pld5	-1.299710358	2.193405959	38.73474728	3.55E-05	0.003341083
Clic6	-1.303642585	1.883426528	17.91687966	0.001399842	0.034024053
S100g	-1.308805389	3.028035845	28.92713651	0.00024329	0.011919778
Aldh1a1	-1.33287065	6.330749785	25.93339481	0.001192234	0.031177762
Ptch2	-1.334022092	3.276116676	78.7201407	8.80E-07	0.000297803
Bhmt2	-1.365938727	0.491714954	28.70983963	0.000143828	0.008344617
F5	-1.425607242	2.333353137	55.06914699	5.98E-06	0.000974358
Sult1c2	-1.431110435	-0.575734115	15.27680299	0.001893631	0.040858815
Stap1	-1.438952366	-1.299251191	16.55487468	0.001407119	0.034119044
Gli1	-1.440724317	5.775706456	93.67196642	2.04E-06	0.000492723
Edil3	-1.441983918	3.08137912	64.73517743	3.06E-06	0.000652565
Cited1	-1.45794662	0.266504901	31.87125023	8.92E-05	0.006007193
Ankfn1	-1.490638657	1.54753146	57.35317538	4.83E-06	0.000857342
Hhip	-1.499833442	0.331464964	19.48827621	0.000748038	0.023734901
Rdh9	-1.511188225	0.428186557	39.199129	3.33E-05	0.003187791
Aldh1a3	-1.522437514	6.108281606	59.46339979	4.00E-05	0.003609554
Nog	-1.541802583	2.400392455	97.71164423	2.64E-07	0.000131227
Has2	-1.562528685	3.518302795	195.0757447	4.74E-09	1.36E-05
Npl	-1.572574153	4.2475159	46.95064181	5.84E-05	0.004485011
Gm4951	-1.596102767	2.266486756	26.8795162	0.000341367	0.01479002
Stra6l	-1.609447431	0.071846539	36.46510377	4.72E-05	0.003914916
Slc38a5	-1.660174449	0.629180336	46.47641779	1.43E-05	0.001712704
Gzmb	-1.681795762	0.539474573	33.58802935	6.98E-05	0.005145673
Ms4a4b	-1.728307134	0.367634143	27.2764851	0.000180908	0.00984825
Klrd1	-1.73866696	-0.213785153	17.86343835	0.001056081	0.028651786
Prokr2	-1.740829715	-0.120920068	25.01041917	0.000264733	0.012584073
Kcnmb2	-1.741844794	-0.085512252	27.40125088	0.000177272	0.009711033
Atp7b	-1.752503617	-0.368963801	22.74103594	0.000397354	0.016223357
Il2rb	-1.803463961	2.102967331	58.05443139	5.36E-06	0.000911017
Bpi	-1.805580524	-0.983187344	18.95095811	0.000835859	0.025401552
Gvin1	-1.821141883	-1.051827269	19.86179179	0.000693287	0.02265019
Qrfprl	-1.843098071	-0.719574213	30.70268956	0.000105962	0.00661092
BC048679	-1.879999472	2.688640393	38.79286054	8.70E-05	0.005919259
Ano2	-1.902885643	3.149007444	48.23876237	3.84E-05	0.003534893
Gm7030	-1.931123685	-1.832500594	17.46435831	0.001148707	0.030466996
Gstm7	-1.971651343	6.691106105	98.92732923	6.14E-06	0.000974358
Ccl5	-1.972288721	-0.089327525	15.98500309	0.001876903	0.040763297
Rdh1	-1.992093605	0.375345583	72.52483196	1.38E-06	0.000377374
Samd3	-2.018766751	-1.670119467	17.34787923	0.001178513	0.030979309
Abca13	-2.104929116	-0.970674928	31.57314589	9.31E-05	0.006157233
Nkg7	-2.136853172	-0.17716162	21.91873093	0.000504783	0.01849519
Ms4a8a	-2.18955936	-1.210028011	16.86628994	0.001311684	0.032857189
Klre1	-2.192376636	-1.118142722	21.88395577	0.000466552	0.017935549
Klrk1	-2.203569944	0.022472164	29.48487078	0.000130356	0.007592553
Cyp26a1	-2.22979281	0.19154395	45.95729093	1.51E-05	0.001786927
Klra5	-2.275046633	-1.30091467	24.82375767	0.000273456	0.012795609
Ncr1	-2.293093611	0.110211801	42.72024116	2.18E-05	0.002364173

Coch	-2.299938169	6.103723609	79.0958763	2.75E-05	0.002797228
Gzma	-2.32008009	0.683788845	58.46030516	4.37E-06	0.00080031
Trpm1	-2.343067422	0.207030477	44.45001142	1.79E-05	0.00203957
Smim41	-2.354540108	2.887253676	106.4405664	3.57E-07	0.000164989
Gzmc	-2.388036049	-0.683589135	33.38560202	7.18E-05	0.005246221
Cadps	-2.502804335	1.416990984	97.46022421	2.68E-07	0.000131227
Sp5	-2.516032224	-0.068165613	53.35411232	7.05E-06	0.001069785
Xcl1	-2.555412554	-0.289460003	44.28513519	1.82E-05	0.002050881
Dio1	-2.591765911	-1.943698217	26.1021351	0.000219729	0.011157852
Cldn2	-2.679846927	-1.329872296	36.42490251	4.75E-05	0.003916198
Fbxo40	-2.714978259	-1.855957234	21.58424806	0.000493983	0.018245612
Vit	-2.715141565	1.998963481	82.57356055	1.37E-06	0.000377374
Klrc1	-2.828166028	-1.61430031	32.27665826	8.41E-05	0.005788268
Foxa2	-2.909124916	0.294640541	85.27126162	5.66E-07	0.000241349
Cd7	-3.017963527	0.035629545	103.6311167	1.89E-07	0.000117829
Egfem1	-3.061983705	-0.711591653	60.00241951	3.81E-06	0.000729619
Galnt14	-3.121886752	-0.099994265	99.77205142	2.35E-07	0.000128768
A2ml1	-3.34692731	0.471652645	80.44544447	7.81E-07	0.000271047
Dleu7	-3.454030353	0.874509025	144.9651555	2.74E-08	3.51E-05
Wif1	-3.475555745	-0.602618152	52.54467667	7.63E-06	0.00111493
Ihh	-3.576625584	1.276366075	112.0614704	1.55E-07	0.000104546
Nos1	-3.69665738	-0.179638815	77.96630628	9.28E-07	0.000303064
Cxcl15	-3.832444558	1.206883406	83.50519308	1.46E-06	0.000385114
Itgbl1	-3.970853653	-0.043575635	78.49754462	8.94E-07	0.000297803
Nalcn	-4.52823801	1.597071405	288.3337011	4.52E-10	5.84E-06
